# Time‐Dependent Therapeutic Effect of *S*‐Ketamine on PTSD Mediated by VTA‐OFC Dopaminergic Neurocircuit

**DOI:** 10.1002/advs.202500805

**Published:** 2025-09-25

**Authors:** Ye Wang, Lei Liu, Jinghao Wang, Jiannan Li, Huiming Li, Rui Wang, Hui Wang, Min Wang, Quanying Liu, Zhongmin Fan, Yunyun Zhang, Xinxin Zhang, Dan Wang, Sa Wang, Rou Xue, Jindong Mao, Min Cai, Pengfei Wei, Hailong Dong, Yumei Wu, Guangchao Zhao

**Affiliations:** ^1^ Department of Anesthesiology and Perioperative Medicine Xijing Hospital The Fourth Military Medical University Xi'an 710032 China; ^2^ Department of Pharmacology School of Pharmacy The Fourth Military Medical University Xi'an 710032 China; ^3^ Key Laboratory of Anesthesiology (The Fourth Military Medical University) Ministry of Education Xi'an 710032 China; ^4^ Shaanxi Provincial Clinical Research Center for Anesthesiology Medicine Xi'an 710032 China; ^5^ Department of Biomedical Engineering Southern University of Science and Technology Shenzhen 518055 China; ^6^ Department of Psychiatry Xijing Hospital The Fourth Military Medical University Xi'an 710032 China; ^7^ School of Biological Science and Medical Engineering State Key Laboratory of Digital Medicine Southeast University Nanjing 211189 China

**Keywords:** PTSD, *S*‐ketamine, TI‐NIBS, VTA dopamine

## Abstract

Current pharmacotherapies for post‐traumatic stress disorder (PTSD) are limited by delayed onset and side effects. Despite ketamine exhibiting rapid relief of the core symptoms of PTSD, its clinical efficacy varies considerably depending on the timing of drug delivery. However, the underlying mechanism remains unclear. In this study, the therapeutic effects of early (day 1) and late (day 7) administration of *S*‐Ketamine on behavioral phenotypes in rodent's models of PTSD are compared. It is observed that early rather than late administration of *S*‐Ketamine significantly ameliorates PTSD symptoms, especially impaired fear extinction. The firing and burst rates of VTA^DA^ neurons consecutively decrease following PTSD modeling and are restored by early *S*‐Ketamine intervention. In particular, VTA^DA^ neurons respond to the conditioned stimuli, mediating the replacement of aversive memory encoding during fear extinction. The inhibition of VTA^DA^‐OFC interrupts the PTSD treatment induced by *S*‐Ketamine. A non‐invasive temporally interfering brain stimulation targeting the OFC is further developed, sensitizing cortical dopaminergic transmission and extending the effective time window of *S*‐Ketamine for anti‐PTSD. Overall, a neural mechanism for the heterogeneous VTA^DA^‐OFC neurocircuit‐mediated time‐dependent therapeutic effect of *S*‐Ketamine is illustrated. In addition, a novel technique is developed to optimize the strategy of ketamine‐assisted psychotherapy for PTSD treatment.

## Graphical abstract

This study reveals that early rather than late administration of *S*‐ketamine can ameliorate PTSD symptoms. This phenomenon is attributed to reversing the progressive inhibition of VTA^DA^ neurons and the function of VTA^DA^‐OFC circuit following stress. Targeted stimulation of the OFC by TI‐NIBS to enhance DA release effectively extends the therapeutic time window for *S*‐ketamine in PTSD treatment.

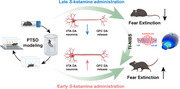

## 1 Introduction

Post‐traumatic stress disorder (PTSD) is a severe psychiatric syndrome characterized by several symptoms following traumatic events, including intrusive memories and re‐experiencing of trauma, avoidance of trauma reminders, negative cognitions and mood, and hyperarousal.^[^ ^1^, ^2^, ^3^ ^]^ Although substantial progress has been made in elucidating the neurobiological mechanisms underlying PTSD, effective treatments remain limited. Current pharmacological treatments, such as selective serotonin reuptake inhibitors and serotonin‐norepinephrine reuptake inhibitors, are limited by their slow onset of action, prolonged treatment duration, and indisposed side effects.^[^ ^4^, ^5^ ^]^ Ketamine, a non‐competitive NMDA receptor antagonist,^[^ ^6^ ^]^ has attracted increasing attention because of its rapid antidepressant effects and potential benefits in PTSD treatment. However, its efficacy remains controversial. A randomized clinical trial (RCT) demonstrated that a 0.5 mg kg^−1^ intravenous infusion of ketamine rapidly attenuates the core symptoms of PTSD.^[^ ^7^ ^]^ Another large matched‐cohort study that enrolled 1158 combat‐injured U.S. service members concluded that ketamine administration during hospitalization for analgesia did not affect the prevalence of PTSD.^[^ ^8^ ^]^ Notably, evidence from basic research has demonstrated that ketamine alleviates stress‐induced behaviors only when administered within a specific reconsolidation window, typically within 6 h of trauma exposure.^[^ ^9^ ^]^ These findings suggest that the therapeutic potential of ketamine in PTSD may vary with drug delivery time.

Considerable evidence indicates that midbrain dopaminergic systems, particularly in the ventral tegmental area (VTA), play a critical role in the development and progression of PTSD.^[^ ^10^, ^11^, ^12^ ^]^ Mechanistically, acute stress dynamically alters neuronal activity in mesolimbic dopaminergic circuits, with VTA dopamine (VTA^DA^) neurons showing prolonged decreases in excitability and burst firing after trauma.^[^ ^13^ ^]^ Ketamine modulates VTA^DA^ neuronal activity and firing patterns to rescue behavioral abnormalities,^[^ ^14^, ^15^, ^16^ ^]^ indicating that these neurons may serve as therapeutic targets for alleviating PTSD symptoms. In addition, the orbitofrontal cortex (OFC), a pivotal prefrontal subregion that functions as a neural integration hub, receives dense dopaminergic projections from VTA neurons and plays a crucial role in modulating adaptive behavioral responses, particularly in fear extinction processes.^[^ ^17^, ^18^, ^19^ ^]^ Considering their functional specificity and clinical accessibility, OFC neural networks have become important targets for neuro‐modulatory therapy for several psychiatric disorders, such as transcranial alternating/direct current stimulation (tACS/DCS) for depression.^[^ ^20^, ^21^ ^]^ Nevertheless, it remains unclear whether the discrepancies in the therapeutic efficacy of ketamine based on delivery time can be attributed to VTA^DA^ neurons and associated neurocircuits.

A major limitation of existing fundamental research on PTSD is the lack of robust and reportable behavioral assessment tools for rodents. Recently, AI‐assisted behavior analysis systems have enabled precise quantification and clustering of spontaneous behaviors in mice. In the present study, we used a hierarchical 3D‐motion learning framework to compare the different effects of early (24 h post‐modeling) and late (7 days post‐modeling) administration of *S*‐ketamine (i.p. 10 mg kg^−1^), an enantiomer of (*R, S*)‐Ketamine, on PTSD behavioral phenotypes in mice that underwent the single prolonged stress and shock (SPS&S) modeling,^[^ ^22^ ^]^ especially for spontaneous behaviors and fear extinction. Furthermore, we used advanced techniques, including in vivo electrophysiology, opto/chemo‐genetic manipulation, and fiber‐photometry recording, to investigate the dynamic changes in VTA^DA^ neurons discharge during PTSD progression, and the underlying neural mechanism of the timing‐dependent therapeutic effect of *S*‐ketamine on PTSD. In addition, we developed a non‐invasive temporally interfering brain stimulation (TI‐NIBS) technique targeting the OFC, which is innervated by VTA^DA^ neurons, to extend the time window of *S*‐ketamine delivery during PTSD treatment. This study aimed to elucidate the neural mechanisms mediating the timing‐dependent effect of *S*‐ketamine on PTSD and provide a novel noninvasive strategy for the optimization of this clinical intervention.

## 2 Results

### 2.1 Early Instead of Late Administration of *S*‐Ketamine Alleviates PTSD‐Like Behavior

**Figure 1:**
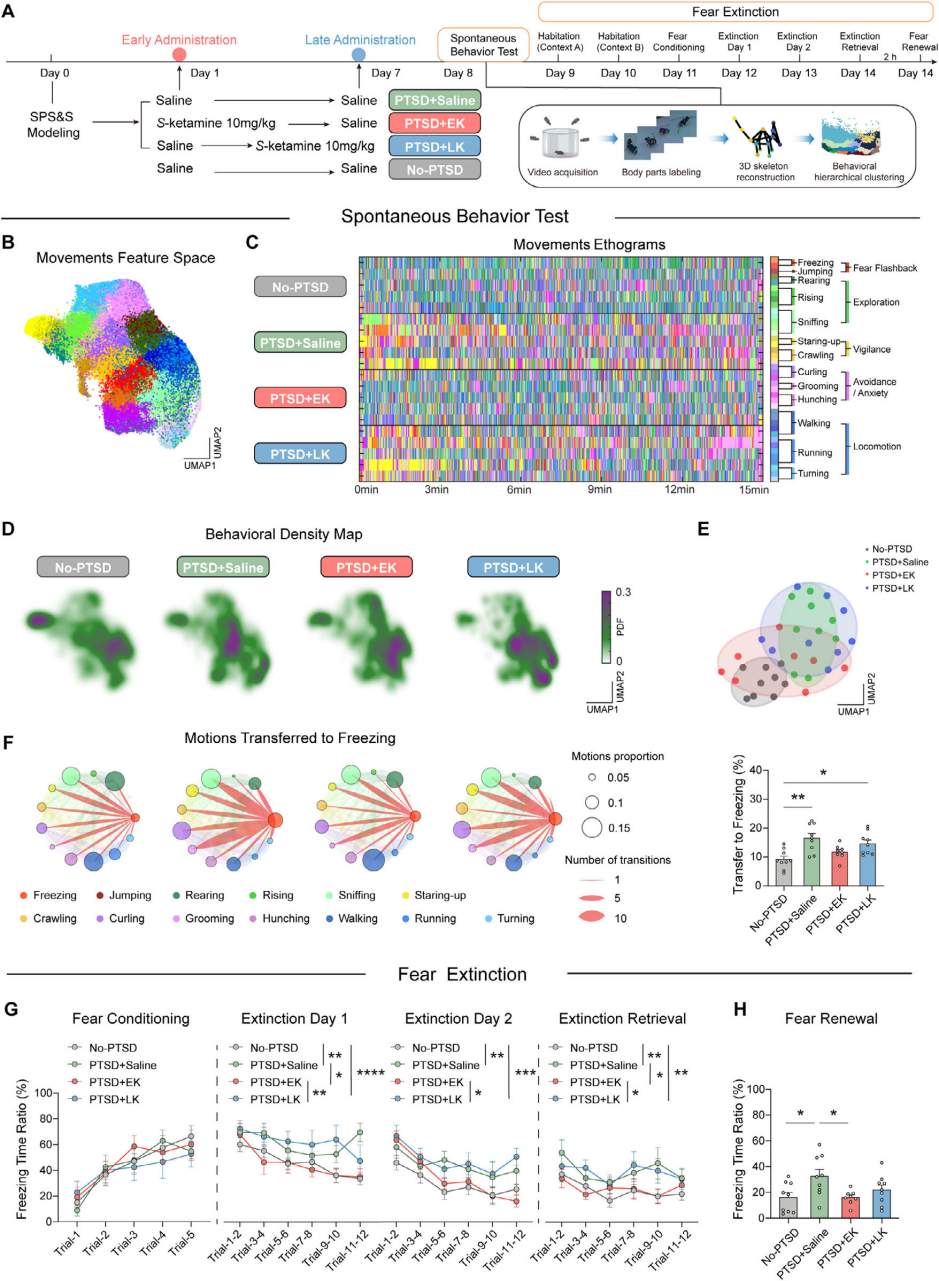
Early instead of late administration of *S*‐Ketamine alleviates PTSD‐like behavior. A) Schematic of drug injection and behavioral tests. B) Spatiotemporal feature space of movements’ components. Each dot on the scatter plot represents a movement bout. The 40 different colors indicate the corresponding 40 movement types. C) Representative ethograms of 40 movements and manually assigning annotations. D) Heatmap of the feature space probability density of each group of behavior segments. Areas with higher probability density (purple) indicate denser behavioral segments in that region, while areas with lower probability density (white) indicate sparser behavioral segments. E) Low‐dimensional representation of the four animal groups generated through UMAP dimensionality reduction. F) Movements transferred to freezing between four groups. The size of the colored circle represents the proportion of the corresponding behavior, and the diameter of the line segment represents the number of transformations. F (3, 31) = 1.514, one‐way ANOVA with *Tukey* test. G) Freezing responses to the CS during fear conditioning (F (3, 155) = 0.2713), extinction (Day 1: F (3, 201) = 9.297; Day 2: F (3, 186) = 8.546), and extinction retrieval (F (3, 186) = 6.691), two‐way repeated measures ANOVA. (H) Freezing responses during fear renewal, F (3, 31) = 2.385, one‐way ANOVA with *Tukey* test. NO‐PTSD, PTSD+Saline, PTSD+LK, *n* = 9 mice, PTSD+EK, *n* = 8 mice. Data are presented as means ± SEM, ^*^ *p* <0.05, ^**^ *p* <0.01, ^***^ *p* <0.001. PDF, probability density function; UMAP, uniform manifold approximation and projection.

To assess the effects of *S*‐ketamine delivered at different time points on PTSD‐like behaviors in mice after SPS&S modeling, the mice received intraperitoneal injections of 10 mg kg^−1^ *S*‐ketamine either on the first day (early *S*‐Ketamine intervention, PTSD+EK group) or seventh day after modeling (late *S*‐ketamine intervention, PTSD+LK group). Spontaneous behaviors were evaluated on day 8 after modeling using the elevated plus maze (EPM) and open field (OFT) tests (**Figure** **1**A). Performances in both the EPM and OFT tests revealed that PTSD modeling induced anxiety‐like behaviors, which manifested as reduced time spent in the open arm and decreased entries to the open arm in the EPM, as well as decreased movement in the center zone and total travel distance in the OFT (Figure S1, Supporting Information). Both early and late *S*‐ketamine interventions alleviated anxiety‐like behaviors.

To achieve accurate and quantitative measurements of spontaneous behaviors, we used an unsupervised 3D‐motion machine‐learning framework. The feature space of the movements was generated using Uniform Manifold Approximation and Projection (UMAP) (Figure 1B). Based on Mouse Ethogram database and previous studies^[^ ^23^, ^24^, ^25^, ^26^, ^27^ ^]^ (Table S1, Supporting Information), we identified 40 movements and categorized them into 13 behavioral characteristics: fear flashbacks (freezing, jumping), exploration (rearing, rising, sniffing), vigilance (staring‐up, crawling), avoidance/anxiety (curling, grooming, hunching), and locomotion (walking, running, turning). This toolbox of behavioral analysis helped us determine the core symptoms of PTSD in mice, similar to the PTSD diagnosis in humans according to the DSM‐V guidelines; the symptoms included intrusion, avoidance, negative alterations in cognition and mood, and changes in arousal and reactivity (Figure 1C; Figure S2A, Supporting Information).

PTSD modeling led to decreased exploratory and locomotion‐related behaviors, increased fear flashbacks, and avoidance/anxiety‐related behaviors; EK intervention reversed these behavioral alterations, whereas LK intervention showed no significant improvement (Figure 1D; Figure S2B–E, Supporting Information). Dimensionality reduction and unsupervised clustering clearly differentiated the PTSD+Saline group (mice that underwent SPS&S modeling and were administered saline) and the No‐PTSD group (mice without PTSD modeling). Individual clustering of the PTSD+EK group was much closer to that of the No‐PTSD group, whereas the PTSD+LK group clustered near the PTSD+Saline group (Figure 1E). Further analysis revealed that PTSD modeling resulted in an 81% increase in transitions to freezing behavior, indicating heightened fear memory flashbacks, whereas the EK intervention resembled the No‐PTSD group. In contrast, the LK intervention group still exhibited a marked increase in transitions (No‐PTSD 9.171 ± 1.113 vs PTSD+Saline 16.6 ± 1.584 vs PTSD+EK 11.73 ± 0.891 vs PTSD+LK 14.62 ± 1.266) (Figure 1F). These results indicate that EK intervention is more effective than LK intervention in mitigating PTSD‐like behaviors and restoring normal behavioral patterns in mice.

In addition, we investigated the time‐dependent effects of *S*‐ketamine administration on fear memory and extinction. Mice were exposed to a 30‐s tone (4500 Hz, 75 dB) as the conditioned stimulus (CS) paired with a 2‐s foot shock (0.6 mA) as the unconditioned stimulus (US). During fear conditioning, there were no significant differences in freezing behavior between the groups, regardless of prior SPS&S exposure or *S*‐Ketamine treatment. However, during fear extinction and fear renewal tests, PTSD‐modeled mice displayed significantly elevated freezing levels, indicating impaired fear extinction. EK intervention effectively reduced freezing levels, whereas LK intervention did not produce similar effects (Figure 1G,H). These results suggest that prior SPS&S exposure does not affect the initial formation of fear memory but impairs fear extinction. EK administration ameliorated this impairment, whereas LK intervention did not provide comparable benefits.

Cluster analysis of the 3D spontaneous behavior profiles across all samples revealed two distinct clusters (Clusters 1 and 2). Cluster 1 comprised 88.9% of subjects in the No‐PTSD group, whereas Cluster 2 comprised mainly PTSD+Saline subjects (88.9%) (Figure S2F, Supporting Information). A statistical comparison of the freezing time ratio during fear memory formation and extinction demonstrated that Cluster 1 exhibited a significantly lower fear response than Cluster 2, aligning with the behavioral phenotypes observed in the No‐PTSD and PTSD+Saline (Figure S2G,H, Supporting Information).

To standardize administration intervals between PTSD+EK and PTSD+LK groups, late ketamine intervention groups were behaviorally tested at matched early‐intervention timepoints (Figure S3A, Supporting Information). The *S*‐Ketamine administration group showed no behavioral improvement compared to saline controls, confirming that the inefficacy of delayed intervention is independent of drug exposure intervals (Figure S3B–F, Supporting Information). Additionally, *S*‐Ketamine administration within 6 h after PTSD modeling also alleviates PTSD‐like behaviors (Figure S4, Supporting Information).

### 2.2 Dynamic Alteration of Excitability and Firing Patterns Among VTA^DA^ Neurons During PTSD Progression

**Figure 2:**
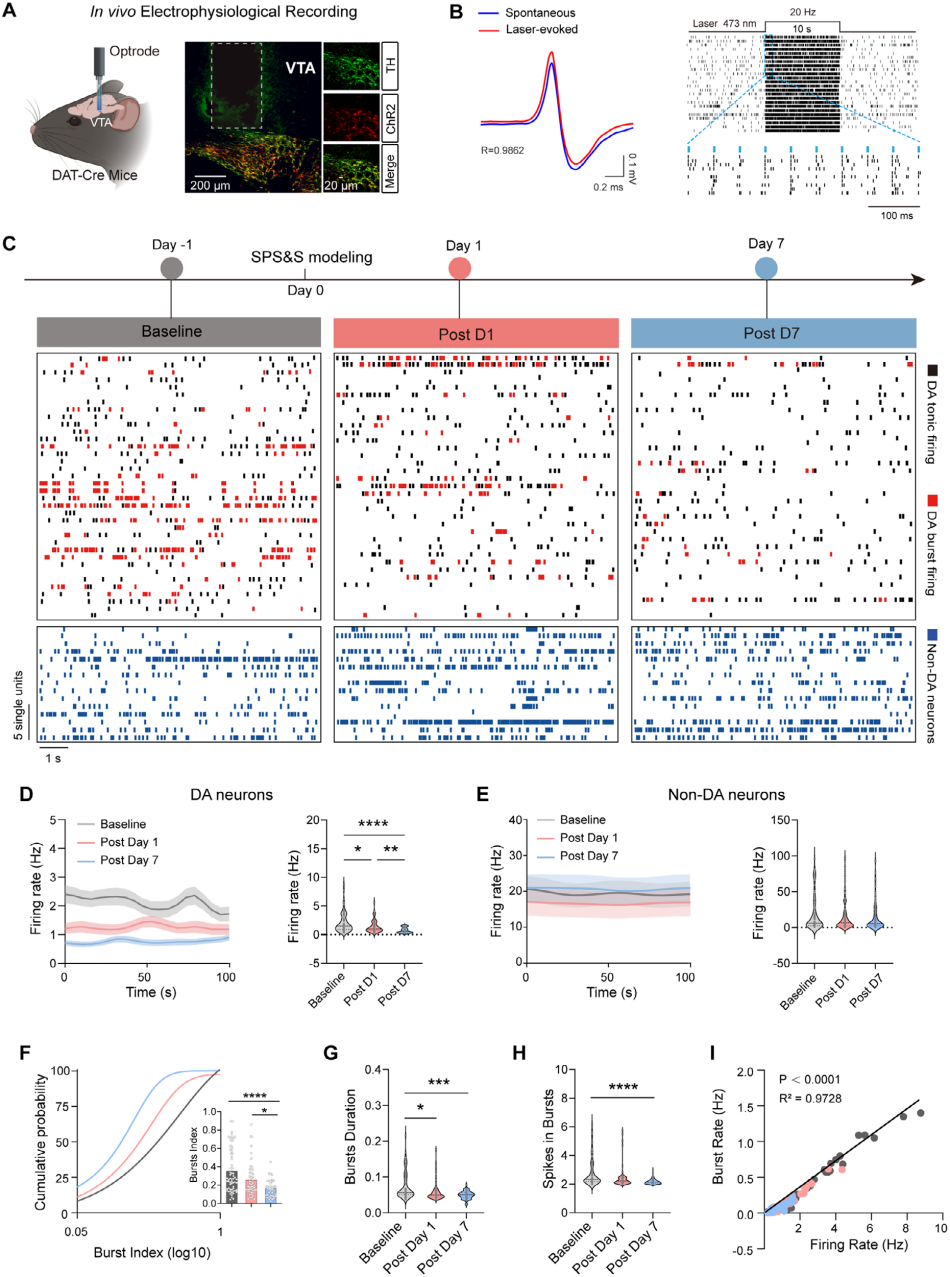
Dynamic alteration of excitability and firing patterns among VTA^DA^ neurons during PTSD progression. A) Schematic of in vivo tetrode implantation (left) and representative image of ChR2 expression and localization of electrode in the VTA (right). B) Waveforms of average spontaneous (blue) and individual laser‐evoked (red) spikes from an identified neuron in the VTA ensemble. (left) and example of a raster plot from a neuron in the VTA ensemble showing consecutive laser stimulation trials at 20 Hz (right). C) Schematic of in vivo electrophysiology recording (top) and representative raster plot of DA and non‐DA neurons. D) Firing rate of DA neurons; *p* <0.0001, *n* = 63 units in baseline, *n* = 49 units in post D1, *n* = 42 units in post D7, *Kruskal–Wallis* test. E) Firing rate of non‐DA neurons; *p* = 0.6196, *Kruskal–Wallis* test, *n* = 73 units in baseline, *n* = 108 units in post D1, *n* = 116 units in post D7. F) Cumulative distribution and comparison of the burst index (the ratio of burst events to the total number of spikes) of DA neurons; F (2, 151) = 3.177, *p* <0.0001; one‐way ANOVA with *Tukey* test. G and H) Bursts duration (*p* = 0.0002) and spikes in bursts (*p* <0.0001) of DA neurons, *Kruskal–Wallis* test. J) The correlation between DA neurons firing rate and burst rate. DA neurons *n* = 63 units in baseline, *n* = 49 units in post D1, *n* = 42 units in post D7. Data are presented as means ± SEM, ^*^ *p* <0.05, ^**^ *p* <0.01, ^***^ *p* <0.001, ^****^ *p* <0.001.

Whole‐brain c‐Fos mapping results revealed that, among the brain regions closely associated with PTSD, only neuronal activity in the VTA exhibited a sustained decrease following model establishment (Figure S5, Supporting Information). To further investigate temporal changes in VTA neuronal activity after PTSD modeling, we used in vivo electrophysiological recordings to monitor neuronal activity at baseline (pre‐modeling) and on days 1 and 7 after PTSD modeling. To rule out potential confounds from chronic electrode implantation artifacts, parallel control experiments were performed in non‐PTSD model mice using identical recording protocols and time points. We transfected AAV2/9‐Ef1a‐DIO‐ChR2‐mCherry into DAT‐Cre mice to selectively label dopamine (DA) neurons, which were, thereafter, activated by 473 nm blue light. Following 20 Hz light stimulation, DA neurons were strongly activated, confirming the specificity of our approach (**Figure** **2**A,B). We recorded spontaneous neural activity in 136 neurons before modeling, 157 neurons on day 1 after modeling, and 158 neurons on day 7 after modeling. Among these, 63, 49, and 42 DA neurons were identified, respectively. Our analysis revealed a significant reduction in DA neuron firing rates on day 1 after modeling, which decreased further on day 7 (Baseline 2.204 ± 0.2258 Hz vs Post Day 1 1.435 ± 0.1604 Hz vs Post Day 7 0.7587 ± 0.07414 Hz) (Figure 2C,D). In contrast, the firing rate of non‐DA neurons showed no significant change over time (Figure 2C–E). Burst firing was defined as previously described.^[^ ^28^ ^]^ The burst index of DA neurons, defined as the ratio of burst events to total spikes, was significantly reduced on day 1 modeling compared to baseline, with an even greater reduction observed on day 7, as well as burst duration (Figure 2F–G). The spikes within bursts in DA neurons were significantly lower on day 7, whereas no significant difference was observed on day 1 compared with baseline (Figure 2H). Besides, control mice exhibited no time‐dependent alterations in firing frequency for either DA or non‐DA neurons and maintained stable burst dynamics across all intervals (Figure S6, Supporting Information), confirming that electrode implantation alone did not drive progressive hypoactivity or associated electrophysiological changes. In addition, the firing rates of VTA^DA^ neurons showed a significant positive correlation with burst firing (Figure 2I). These findings suggest a progressive, time‐dependent suppression of DA neuronal activity following PTSD modeling, whereas non‐DA neurons remained unaffected.

### 2.3 Early *S*‐Ketamine Intervention Facilitates Fear Extinction by Reversing VTA^DA^ Neuronal Hypoactivity in PTSD Progression

**Figure 3:**
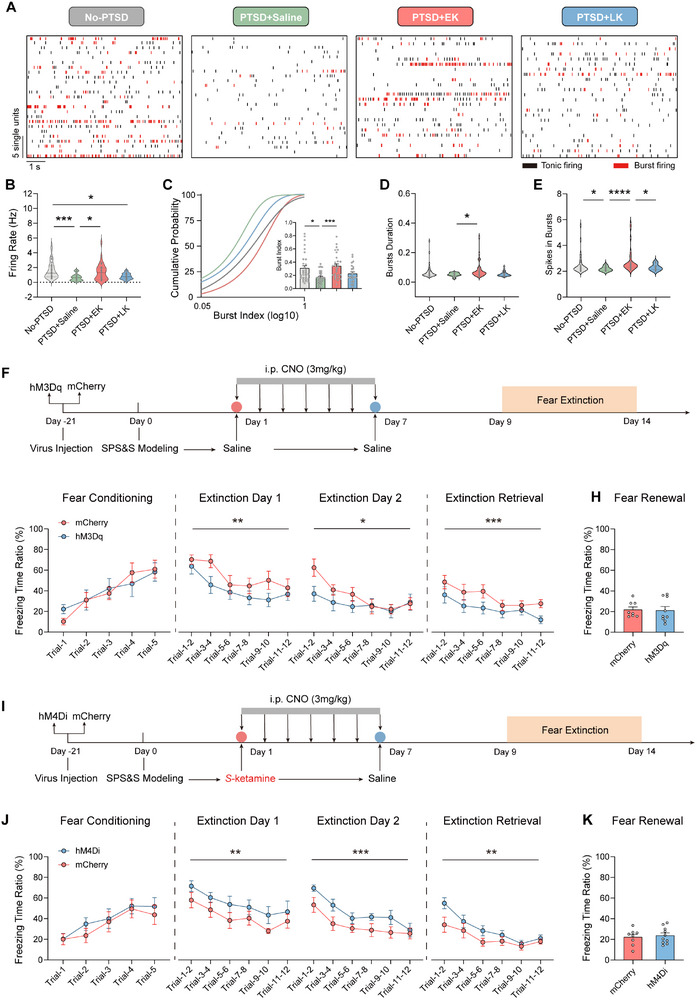
Early *S*‐ketamine intervention facilitates fear extinction by reversing VTA^DA^ neuronal hypoactivity in PTSD progression. A) Representative raster plot of DA neurons. B–E) Firing rate (B, *p* = 0.0001, *Kruskal–Wallis* test), cumulative distribution and comparison of the burst index (the ratio of burst events to the total number of spikes) (C, F (3, 110) = 0.9053, *p* = 0.0004, one‐way ANOVA with *Tukey* test), bursts duration (D, *p* = 0.0159, *Kruskal–Wallis* test) and spikes in bursts (E, *p* <0.0001, *Kruskal‐Wallis* test) of DA neurons. *n* = 32 units in no‐PTSD group, *n* = 29 units in PTSD+Saline group, *n* = 29 units in PTSD+EK group, and *n* = 24 units in PTSD+LK group; *n* = 6 mice. F) Schematic of chemogenetic excitation behavioral tests. G) Freezing responses to the CS during fear conditioning (F (1, 75) = 0.01719, *p* = 0.896), extinction (Day 1: F (1, 90) = 8.314, *p* = 0.0049; Day 2: F (1, 90) = 4.333, *p* = 0.0402) and extinction retrieval (F (1, 90) = 11.90, *p* = 0.0009); mCherry, *n* = 8 mice; hM3Dq, *n* = 9 mice; two‐way repeated measures ANOVA. H) Freezing responses during fear renewal; mCherry, *n* = 8 mice; hM3Dq, *n* = 9, mice; t (15) = 0.1613, *p* = 0.874; unpaired *t* test. I) Schematic of chemogenetic inhibition behavioral tests. J) Freezing responses to the CS during fear conditioning (F (1, 80) = 1.012, *p* = 0.3174), extinction (Day 1: F (1, 96) = 9.045, *p* = 0.0034; Day 2: F (1, 96) = 14.56, *p* = 0.0002) and extinction retrieval (F (1, 96) = 9.629, *p* = 0.0025); mCherry, *n* = 8 mice; hM4Di, *n* = 10 mice; two‐way repeated measures ANOVA. K) Freezing responses during fear renewal; mCherry, *n* = 8 mice; hM4Di, *n* = 10 mice; t (16) = 0.4292, *p* = 0.6735; unpaired *t* test. Data are presented as means ± SEM, ^*^ *p* <0.05, ^**^ *p* <0.01, ^***^ *p* <0.001.

Using in vivo electrophysiological recordings, we assessed the effects of *S*‐Ketamine on VTA^DA^ neuronal activity at different time points after PTSD modeling. On day 8 after modeling, prior to the initiation of fear conditioning, VTA^DA^ neurons in the PTSD+EK group remained persistently excited, with firing rates, burst index, burst duration, and spikes in bursts similar to those in the No‐PTSD group. In contrast, VTA^DA^ neuronal activities in the PTSD+LK and PTSD+Saline groups were lower (**Figure** **3**A–E). These findings indicate that EK intervention appears to sustain enhanced dopaminergic neuron excitability, whereas delayed intervention fails to produce a similar effect, highlighting the importance of EK intervention for restoring VTA^DA^ function.

Specifically, we examined the immediate effects of intraperitoneal injection of *S*‐Ketamine on days 1 and 7 after modeling by recording 30 min of baseline activity before the intraperitoneal administration of 10 mg kg^−1^ *S*‐Ketamine and continued recording for an additional 30 min (Figure S7A, Supporting Information). On day 1 after modeling, the results demonstrated a significant increase in the firing rate of VTA^DA^ neurons following ketamine administration compared to pre‐administration levels (Figure S7B–D, Supporting Information). In addition, both the burst index and spikes in bursts were significantly elevated, indicating increased neuronal excitability. In contrast, when the same dose was administered on day 7 after modeling, no significant changes in firing rates or burst activity were observed (Figure S7E–H, Supporting Information). In addition, RNA sequencing of VTA tissues from PTSD‐modeled mice at 1 and day post‐trauma (with sham controls processed in parallel) identified 232 synaptic‐related differentially expressed genes (DEGs), supported by Gene Ontology (GO) enrichment in synaptic components (Figure S7I,J, Supporting Information). Subsequent qRT‐PCR validation revealed transient upregulation of NMDA and AMPA receptor subtypes at 1‐day post‐PTSD, followed by a return to baseline levels by day (Figure S7K, Supporting Information). These dynamic changes in NMDA and AMPA receptor expression may mediate the differential responsiveness of VTA^DA^ neurons to *S*‐Ketamine interventions.

To clarify the causal relationship between VTA^DA^ neuronal excitability and fear extinction, we used DREADDs to modulate VTA^DA^ neurons in DAT‐Cre mice. Specifically, mice were transfected with AAV2/9‐Ef1α‐DIO‐hM3Dq‐mCherry or AAV2/9‐Ef1α‐DIO‐mCherry bilaterally in the VTA. Starting on the first day after PTSD modeling, clozapine N‐oxide (CNO; 3 mg kg^−1^) was administered intraperitoneally for seven consecutive days. From the 9th to the 14th day after modeling, fear conditioning and extinction tests were conducted (Figure 3F). The results showed that activating VTA^DA^ neurons during PTSD progression did not affect fear memory formation but significantly facilitated fear extinction without influencing fear renewal (Figure 3G,H). In addition, we examined the effects of VTA^DA^ neuronal inhibition by transfecting DAT‐Cre mice with AAV2/9‐Ef1α‐DIO‐hM4Di‐mCherry or AAV2/9‐Ef1α‐DIO‐mCherry in the VTA. Mice received concurrent intraperitoneal injections of CNO (3 mg kg^−1^) and *S*‐Ketamine starting on the first day after PTSD modeling. CNO administration was continued for 7 days (Figure 3I). The results revealed that inhibiting VTA^DA^ neuronal activity diminished the effect of *S*‐Ketamine in promoting fear extinction, without affecting fear memory formation and fear renewal (Figure 3J,K). These results collectively suggest that VTA^DA^ neurons are involved in regulating the extinction of fear memory but do not affect fear renewal, and that EK intervention promotes fear extinction during PTSD progression by sustaining VTA^DA^ neuronal excitability.

### 2.4 Early *S*‐Ketamine Intervention Induces Hypersensitivity of VTA^DA^ Neurons Promoting Fear Extinction

**Figure 4:**
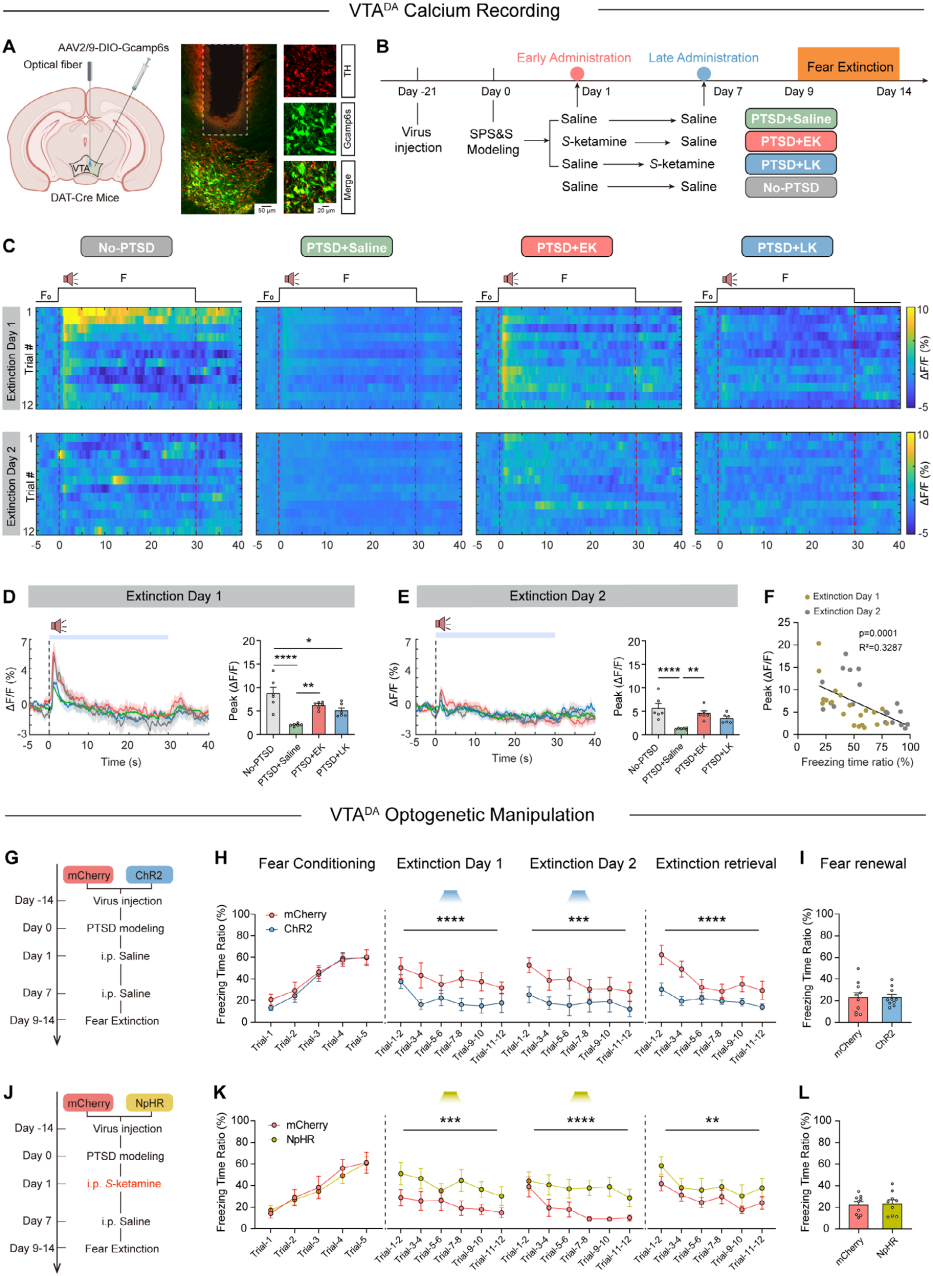
Early *S*‐ketamine intervention induces hypersensitivity of VTA^DA^ neurons, promoting fear extinction. A) Schematic of optical fiber implantation, viral injection (left), and representative image of Gcamp6s and tyrosine hydroxylase (TH) fluorescence co‐expression in the VTA and localization of fiber in the VTA (right). B) Schematic of calcium recording. C) Heatmap of calcium signals aligned to the onset of the CS during fear extinction in each trial. D) Average calcium signals in four groups (left) and the peak calcium signals during the CS for each trial across four groups (right) during extinction day 1. *n* = 5, F (3, 96) = 11.54, *p* <0.0001); two‐way repeated measures ANOVA. E) Average calcium signals in four groups (left) and the peak calcium signals during the CS for each trial across four groups (right) during extinction day 2; *n* = 5, F (3, 96) = 7.551, *p* = 0.0001); two‐way repeated measures ANOVA. F) Correlation between the average freezing time ratio and the average calcium peak for each mouse. G) Schematic of optogenic excitation during extinction. H) Freezing responses to the CS during fear conditioning (F (1, 90) = 0.5126, *p* = 0.4759), extinction (Day 1: F (1, 108) = 17, *p* <0.0001; Day 2: F (1, 108) = 14.22, *p* = 0.0003) and extinction retrieval (F (1, 108) = 27.48, *p* <0.0001); *n* = 10, two‐way repeated measures ANOVA. I) Freezing responses during fear renewal; *n* = 10, t (18) = 0.03649, *p* = 0.9713; unpaired t test. J) Schematic of optogenic inhibition during extinction. K) Freezing responses to the CS during fear conditioning (F (1, 80) = 0.2124, *p* = 0.6462), extinction (Day 1: F (1, 96) = 15.81, *p* = 0.0001; Day 2: F (1, 96) = 23.80, *p* <0.0001) and extinction retrieval (F (1, 96) = 7.393, *p* = 0.0078); *n* = 9, two‐way repeated measures ANOVA. L) Freezing responses during fear renewal, *n* = 9; t (16) = 0.2481, *p* = 0.8072; unpaired *t*‐test. Data are presented as means ± SEM, ^**^ *p* <0.01, ^***^ *p* <0.001, ^****^ *p* <0.0001.

To examine the effect of EK intervention on VTA^DA^ neuronal activity during fear extinction, we used fiber photometry to monitor calcium signals in vivo while the mice underwent fear extinction (**Figure** **4**A). Mice underwent 24 trials of continuous 30s CS presentations (Figure 4B). The 5s preceding each CS onset was defined as the pre‐CS baseline.

During fear extinction, the calcium activity of VTA^DA^ neurons in PTSD+Saline mice was significantly lower than that in No‐PTSD mice during CS onset. However, EK intervention restored the responsiveness of VTA^DA^ neurons to CS, which was comparable to that of the No‐PTSD group, indicating a normalization of neuronal function. In contrast, LK intervention did not increase VTA^DA^ responsiveness, which remained at a relatively low level, similar to that in the PTSD+Saline group (Figure 4C–E). Notably, the overall responsiveness of VTA^DA^ neurons was reduced on the second day of fear extinction. In addition, we observed a negative correlation between the excitability of VTA^DA^ neurons and freezing levels (Figure 4F). These results indicate that PTSD modeling reduces VTA^DA^ neuronal responsiveness to aversive stimuli. Furthermore, EK intervention enhanced VTA^DA^ neuronal responsiveness, facilitating extinction, whereas LK intervention had a limited effect on neuronal activity. These findings are consistent with the findings presented above, suggesting that *S*‐Ketamine intervention in the early period after severe stress could more effectively prevent PTSD‐induced decline in the function of VTA^DA^ neuron.

To directly examine the causal relationship between VTA^DA^ neuronal activity and fear extinction, we used the bidirectional modulation of VTA^DA^ neurons. First, DAT‐Cre mice were transfected with AAV2/9‐Ef1α‐DIO‐ChR2‐mCherry or AAV2/9‐Ef1α‐DIO‐mCherry in the VTA, followed by implantation of optical fibers (Figure 4G). This viral and transgenic approach successfully labeled VTA dopaminergic neurons, as confirmed through histological analysis (Figure S8A, Supporting Information). Optogenetic stimulation was applied using a protocol (473 nm, 20 Hz bursts, 20 ms pulses for 250 ms, delivered every 5 s) during CS presentations in extinction training. In vitro whole‐cell patch recordings verified that this protocol effectively activated VTA^DA^ neurons (Figure S8C, Supporting Information). There was no significant difference in freezing time ratio between the ChR2 and mCherry groups during fear memory encoding. However, opto‐activation of VTA^DA^ neurons during CS onset significantly reduced the freezing time ratio in the ChR2 group compared to that in the control group. Moreover, freezing responses in the ChR2 group remained significantly lower than those in the mCherry group during extinction retrieval (Figure 4H). Notably, the activation of VTA^DA^ neurons had no effect on the process of fear memory renewal.

To verify the necessity of activating VTA^DA^ neurons in early *S*‐Ketamine intervention to mediate the facilitation of fear extinction, we injected the mice *S*‐Ketamine intraperitoneally (10 mg kg^−1^) on day 1 after PTSD modeling and subsequently inhibited VTA^DA^ neurons during fear extinction (Figure 4J). Further, we transfected the VTA of DAT‐Cre mice with AAV2/9‐Ef1α‐DIO‐NpHR3.0‐mCherry or AAV2/9‐Ef1α‐DIO‐mCherry. In vitro whole‐cell patch recordings showed that continuous 594 nm yellow light stimulation effectively inhibited VTA^DA^ neuronal activity (Figure S8B, Supporting Information). Opto‐inhibition of the VTA^DA^ neurons was applied during CS presentations. No significant differences were observed between the NpHR and mCherry groups during fear conditioning. However, VTA^DA^ neuronal inhibition led to a longer freezing time in the NpHR group than in the control group during fear extinction and retrieval. Notably, such opto‐manipulation did not affect the promotion of fear renewal ability induced by *S*‐Ketamine intervention (Figure 4K,L).

### 2.5 Activation of VTA^DA^‐OFC by the Early Administration of *S*‐Ketamine Rescues the Impairment of Fear Extinction After PTSD

**Figure 5:**
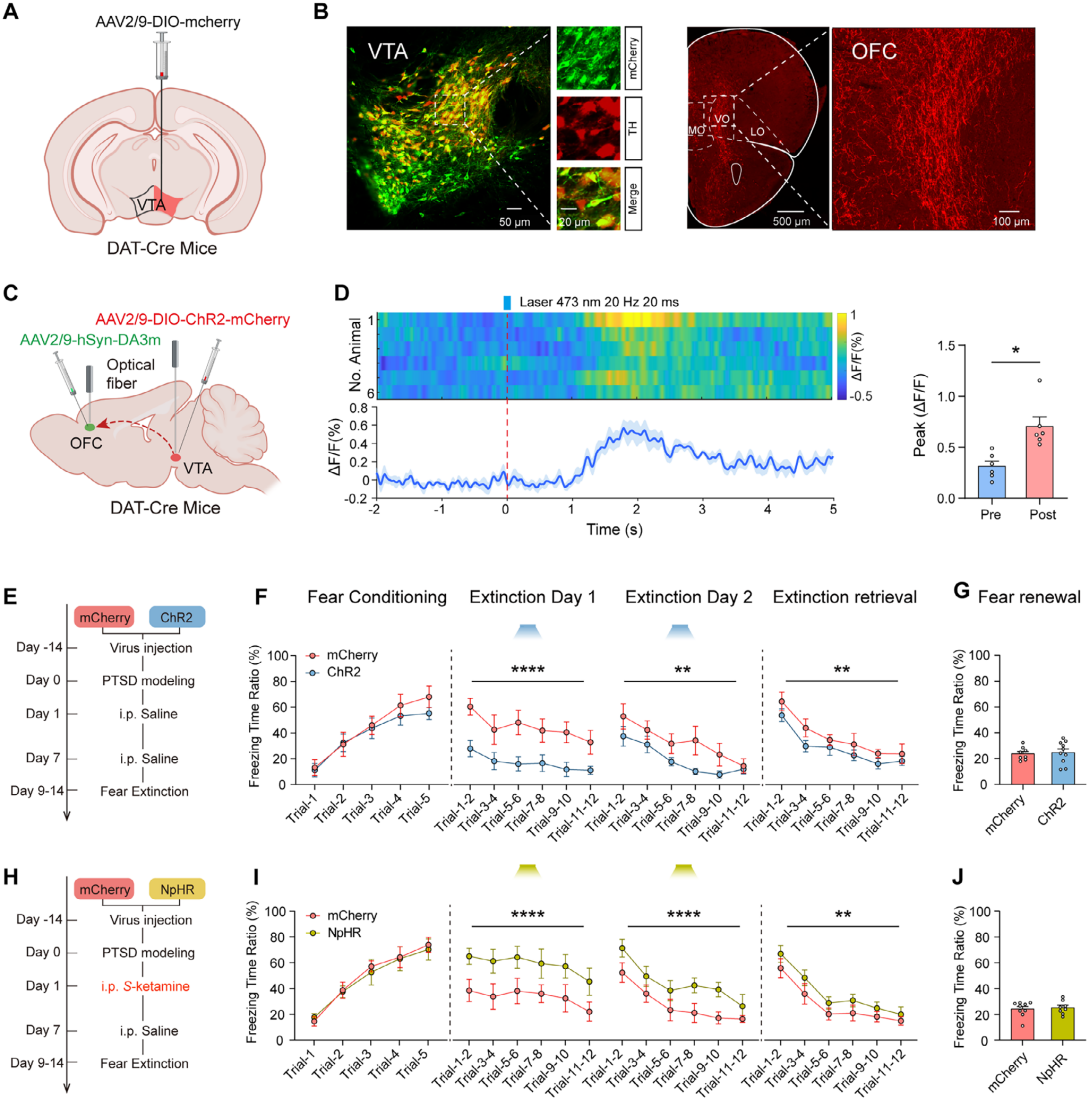
Activation of VTA^DA^‐OFC by early administration of *S*‐ketamine rescues fear extinction impairment after PTSD. A) Schematic of AAV injection to observe VTA^DA^ neuronal projections. B) Representative image of mCherry and tyrosine hydroxylase (TH) fluorescence co‐expression in the VTA (left) and mCherry expression in the OFC region (right). C) Schematic of viral infection and optical fiber implantation to observe changes in DA neurotransmitter levels within the OFC following activation of VTA^DA^ neurons. D) Average calcium signals and heatmap of calcium signals in each mouse (left) and the peak during activation of VTA^DA^ neurons (right); *n* = 6, W = 21, *p* = 0.0313, paired *Wilcoxon* test. E) Schematic of optogenetic excitation of the VTA^DA^‐OFC circuit during extinction. F) Freezing responses to the CS during fear conditioning (F (1, 80) = 1.105, *p* = 0.2963), extinction (Day 1: F (1, 96) = 42.65, *p* <0.0001; Day 2: F (1, 96) = 8.793, *p* = 0.0038) and extinction retrieval (F (1, 96) = 7.430, *p* = 0.0076); mCherry, *n* = 8 mice; ChR2, *n* = 10 mice; two‐way repeated measures ANOVA. G) Freezing responses during fear renewal; mCherry, *n* = 8 mice; *n* = 10 mice; t (16) = 0.2681, *p* = 0.7921, unpaired *t* test. H) Schematic of optogenetic inhibition of the VTA^DA^‐OFC circuit during extinction. I) Freezing responses to the CS during fear conditioning (F (1, 75) = 0.1260, *p* = 0.7237), extinction (Day 1: F (1, 90) = 22.81, *p* <0.0001; Day 2: F (1, 90) = 17.88, *p* <0.0001) and extinction retrieval (F (1, 90) = 7.303, *p* = 0.0082); mCherry, *n* = 9 mice; NpHR, *n* = 8 mice; two‐way repeated measures ANOVA. J) Freezing responses during fear renewal; mCherry, *n* = 9 mice; NpHR, *n* = 8 mice; *p* = 0.9829, unpaired Mann‐Whitney *U* test. Data are presented as means ± SEM, ^*^ *p* <0.05, ^**^ *p* <0.01, ^****^ *p* <0.0001.

To clarify the function of the downstream regions of VTA^DA^ neurons that mediate fear extinction, we injected AAV‐mCherry into the VTA of DAT‐Cre mice to label VTA^DA^ neurons and identify their projections to the cortex (**Figure** **5**A). Our findings revealed that among the VTA^DA^ neuronal projections to the frontal cortex, 29.42% targeted the ventrolateral orbital subregion of the OFC (Figure 5B; Figure S9A,B, Supporting Information). In addition, retrograde tracing using pRV‐CAG‐EGFP virus injected into the OFC of C57 mice demonstrated that 3.75% of DA neurons in the VTA project to the OFC. Notably, among all VTA neurons projecting to the OFC, 38.46% were DA neurons, while 61.54% were non‐DA neurons (Figure S9C–E, Supporting Information). Moreover, opto‐stimulation of VTA^DA^ neurons triggered a significant release of dopamine in the OFC (Figure 5C,D). To determine whether the activation of the VTA^DA^‐OFC pathway could ameliorate the deficits in fear extinction observed in PTSD mice, we transfected the VTA of mice with AAV2/9‐Ef1α‐DIO‐ChR2‐mCherry or AAV2/9‐Ef1α‐DIO‐mCherry, followed by the implantation of optical fibers in the OFC. This allowed us to optogenetically modulate the VTA^DA^ terminals in the OFC during fear extinction. No difference in the freezing time was observed between the ChR2 and mCherry groups during fear conditioning. However, opto‐stimulation of the VTA^DA^ terminals in the OFC significantly reduced freezing responses during fear extinction. This reduction persisted during extinction retrieval (Figure 5E–G), indicating that activation of the VTA^DA^ ‐OFC circuit promotes fear extinction in mice with PTSD.

Subsequently, to validate the role of VTA^DA^‐OFC circuit in *S*‐Ketamine facilitated fear extinction, we conducted specific inhibition in this circuit using AAV2/9‐Ef1α‐DIO‐NpHR3.0‐mCherry transfection and stimuli, following the early administration of S‐Ketamine (i.p.) during the CS presentation (Figure 5H). In accordance with previous findings, the improved fear extinction and retrieval function resulting from EK intervention was prevented by the inhibition of the VTA^DA^‐OFC circuit (Figure 5I,J).

**Figure 6:**
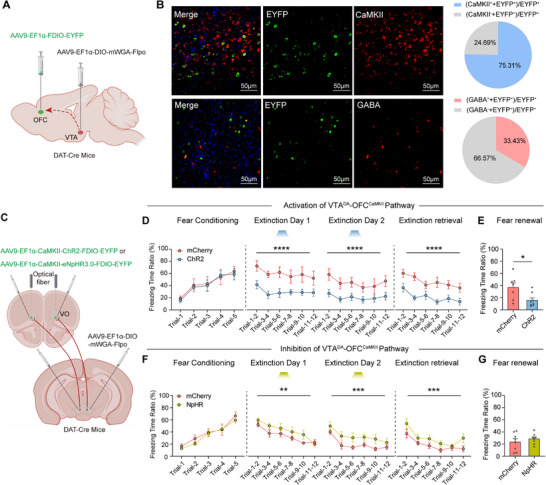
Activation of VTA^DA^ projection‐innervated neurons in OFC by early administration of *S*‐Ketamine rescues fear extinction impairment after PTSD. A) Schematic of viral infection to anterograde tracing. B) Representative micrograph and quantification of VTA^DA^ projection‐innervated neurons in OFC, EYFP labels VTA^DA^ projection‐innervated neurons in OFC, *n* = 3. C) Schematic of viral infection and optical fiber implantation. D) Freezing responses to the CS during fear conditioning (F (1, 65) = 0.1705, *p* = 0.681), extinction (Day 1: F (1, 78) = 40.76, *p* <0.0001; Day 2: F (1, 78) = 31.44, *p* <0.0001) and extinction retrieval (F (1, 78) = 50.49, *p* <0.0001); mCherry, *n* = 7 mice, ChR2, *n* = 8 mice; two‐way repeated measures ANOVA. E) Freezing responses during fear renewal; mCherry, *n* = 7 mice; *n* = 8 mice; *U* = 7, *p* = 0.014, unpaired Mann‐Whitney *U* test. F) Freezing responses to the CS during fear conditioning (F (1, 70) = 1.278, *p* = 0.2622), extinction (Day 1: F (1, 84) = 7.220, *p* = 0.0087; Day 2: F (1, 84) = 14.94, *p* = 0.0002) and extinction retrieval (F (1, 84) = 12.26, *p* = 0.0007); mCherry, *n* = 8 mice; NpHR, *n* = 8 mice; two‐way repeated measures ANOVA. G) Freezing responses during fear renewal; mCherry, *n* = 8 mice; NpHR, n = 8 mice; *p* = 0.4219, t (14) = 0.8274, unpaired t test. Data are presented as means ± SEM, ^*^ *p* <0.05, ^**^ *p* <0.01, ^***^ *p* <0.001, ^****^ *p* <0.0001.

To determine the specific neuronal subtypes in the OFC that form synaptic connections with VTADA neurons, we injected the anterograde trans‐synaptic tracer AAV9‐DIO‐mWGA‐Flop into the VTA and AAV9‐FDIO‐EYFP into the OFC of DAT‐Cre mice. Immunofluorescence staining revealed that the majority (75.31%) of anterogradely labeled cells in the OFC colocalized with glutamatergic (CaMKIIα‐positive) neurons, with minimal (33.43%) colocalization observed with GABAergic (GABA‐positive) neurons (**Figure** **6**A,B). To specifically optogenetically manipulate the VTA^DA^‐OFC^CaMKII^ pathway, we injected AAV9‐DIO‐mWGA‐Flop into the VTA and either AAV9‐CaMKII‐ChR2‐FDIO‐EYFP or AAV9‐CaMKII‐eNpHR‐FDIO‐EYFP into the OFC of DAT‐Cre mice. Optogenetic activation of this pathway during extinction training significantly rescued fear extinction deficits in PTSD‐model mice. Conversely, inhibition of the pathway impaired the efficacy of S‐Ketamine, demonstrating its necessity for the drug's behavioral effects (Figure 6C–G). Collectively, these results demonstrate that EK intervention facilitates fear extinction by activating the VTA^DA^‐OFC^CaMKII^.

### 2.6 TI‐NIBS Enhances Dopaminergic Release in OFC Extending the Therapeutic Time Window of *S*‐Ketamine for PTSD Treatment

**Figure 7:**
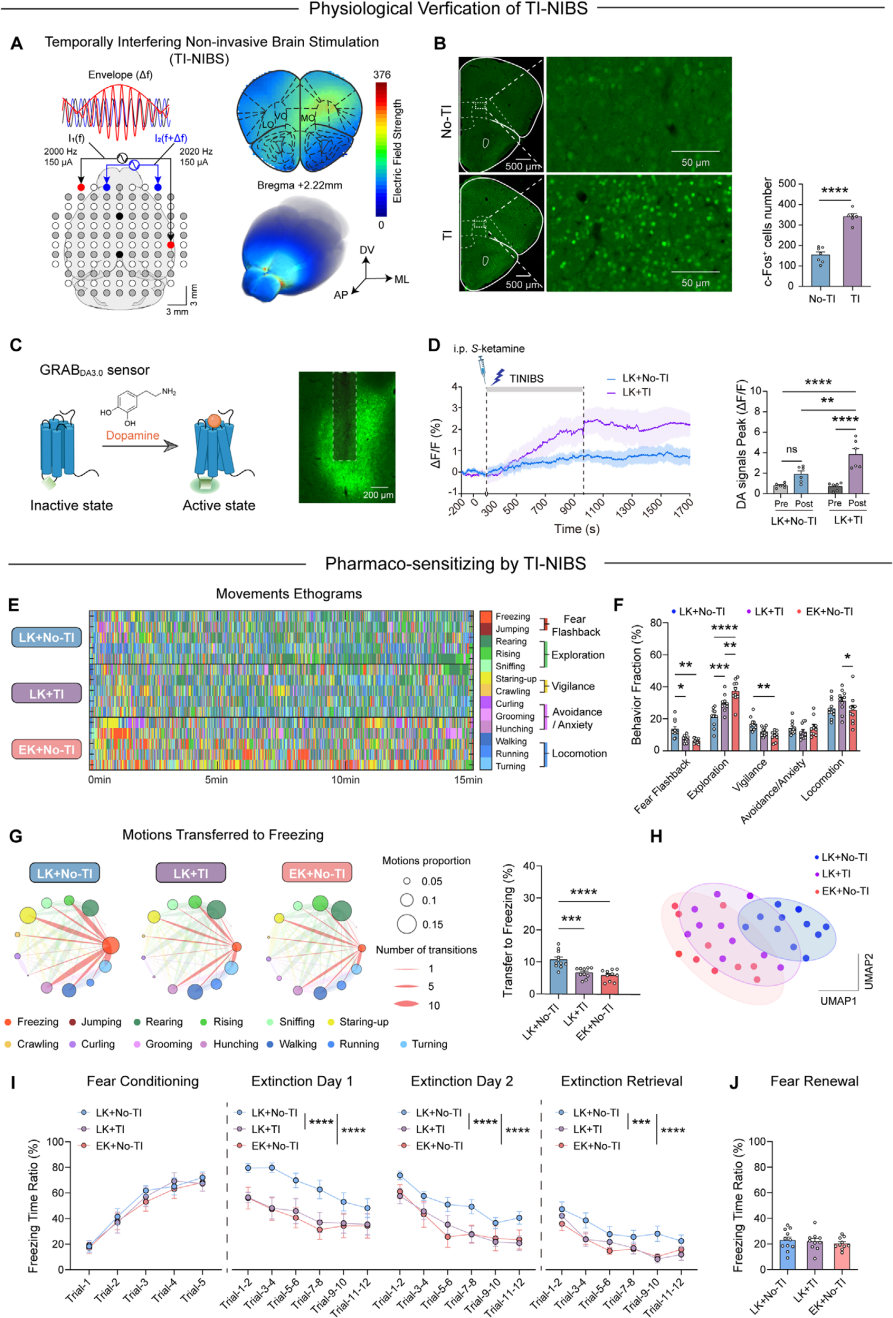
TI‐NIBS enhances dopaminergic release in OFC extending the therapeutic time window of *S*‐ketamine for PTSD treatment. A) Schematic representation of TI‐NIBS electrode placement and its mechanism of action (left) and simulation diagram of the stimulation area (right). B) Representative images showing c‐Fos expression in OFC in No‐TI and TI groups (left) and number of cells immunoreactive for c‐Fos in OFC; No‐TI, *n* = 7 mice; TI, *n* = 6 mice; t (11) = 9.505, *p* <0.0001, unpaired *t*‐test. C) Schematic of the mechanism of the GRAB_DA3.0_ sensor (left) and representative images of its expression in OFC (right). D) Average DA signals in two groups, with *S*‐ketamine injection and TI‐NIBS at time point 0 s (left) and the peak calcium signals before and after drug administration between the two groups; *n* = 6, F (1, 20) = 7.380, *p* = 0.0133, two‐way ANOVA with *Tukey* test. E) Representative ethograms of 13 movements. F) The fraction of five behavioral categories; F (4, 140) = 96.33, *p* <0.0001, two‐way repeated measures ANOVA. G) Movements transferred to freezing between three groups; F (2, 28) = 0.7183, *p* <0.0001, one‐way ANOVA. H) Low‐dimensional representation of the four animal groups generated through UMAP dimensionality reduction. I) Freezing responses to the CS during fear conditioning (F (2, 140) = 0.5189, *p* = 0.5963), extinction (Day 1: F (2, 168) = 20.62, *p* <0.0001; Day 2: F (2, 168) = 14.79), *p* <0.0001, and extinction retrieval (F (2, 168) = 11.59, *p* <0.0001); two‐way repeated measures ANOVA. J) Freezing responses during fear renewal; F (2, 28) = 1.415, *p* = 0.6848, one‐way ANOVA with *Tukey* test. LK+No‐TI, *n* = 11 mice; LK+ TI, *n* = 10 mice; EK+No‐TI, *n* = 10 mice. Data are presented as means ± SEM, ^**^ *p* <0.01, ^***^ *p* <0.001, ^****^ *p* <0.0001.

Given the importance of the delivery time of *S*‐ketamine, we investigated whether combining LK intervention with TI stimulation, an emerging non‐invasive brain stimulation technique, could extend the therapeutic time window for *S*‐ketamine in PTSD treatment. We administered intraperitoneal *S*‐ketamine on day 7 after PTSD modeling and simultaneously applied 20 min of TI‐NIBS (150 µA, 20 Hz) to OFC (**Figure** **7**A). To verify whether TI‐NIBS specifically activated neurons in the OFC, immunofluorescence staining was performed after TI‐NIBS to quantify the expression of c‐Fos, an immediate early gene and a marker of neuronal activation. The results demonstrated that TI‐NIBS specifically increased neuronal activity in the OFC, whereas the adjacent regions exhibited minimal or nonspecific activation (Figure 7B; Figure S10A,B, Supporting Information). To monitor real‐time changes in DA release in the OFC during ketamine administration and TI stimulation, we injected GRABDA3.0 virus into the OFC (Figure 7C). The combination of LK intervention and TI‐NIBS significantly enhanced dopamine release in the OFC (Figure 7D), demonstrating a potential mechanism for this enhanced therapeutic effect.

A series of behavioral assessments was conducted to evaluate the effects of this combined intervention. First, compared to mice in the LK+No‐TI group, mice that received LK intervention combined with TI‐NIBS (LK+TI group) showed a significantly reduced behavioral probability of fear flashbacks and increased exploration‐related behaviors. In addition, TI‐NIBS markedly reduced the spontaneous motions transferred to freezing (LK+No‐TI 10.7 ± 0.8074 vs LK+TI 6.595 ± 0.5107 vs EK+No‐TI 5.784 ± 0.55). These therapeutic effects were comparable to those observed in the EK intervention group (Figure 7E–G; Figure S10C–G, Supporting Information). Unsupervised clustering further demonstrated that LK combined with TI stimulation was more closely associated with EK intervention (Figure 7H). The results of the fear conditioning test showed that this combined modification significantly improved fear extinction, which could not be induced by LK intervention alone (Figure 7I,J). Consistent with our previous findings regarding the activation of the VTA^DA^‐OFC pathway, TI stimulation did not alter freezing responses in the fear renewal test (Figure 7K). Overall, these findings suggest that TI stimulation successfully sensitized the neuronal response to *S*‐Ketamine and restored the therapeutic effects in PTSD when *S*‐Ketamine was administered late. To further investigate the effect of TI‐NIBS alone on the fear extinction deficits, mice received intraperitoneal saline followed immediately by TI‐NIBS on day 7 post‐SPS&S modeling (Saline+TI group). The results show that TI‐NBS alone (Saline+TI group) partially alleviated fear extinction deficits in the PTSD model. However, this effect was significantly weaker than that of the LK+TI group (late ketamine + TI stimulation) (Figure S10H,I, Supporting Information). These results demonstrate that at the late stage of PTSD, while TI‐NBS contributes to rescue, optimal efficacy requires synergistic enhancement by *S*‐Ketamine.

## 3 Discussion

In this study, we investigated the time‐dependent therapeutic effects of *S*‐Ketamine on PTSD‐like behavior and its underlying neural mechanisms. We observed that early *S*‐Ketamine administration during PTSD progression significantly alleviated abnormal spontaneous behaviors and deficits in fear extinction, whereas late *S*‐Ketamine administration only partially mitigated anxiety‐like behaviors. Specifically, VTA^DA^ neurons displayed a consecutive reduction in firing and burst rates of discharge following PTSD modeling, which was restored by early *S*‐Ketamine intervention but not by late intervention. In particular, VTA^DA^ neurons responded to conditioned stimuli, mediating the replacement of aversive memory encoding with the original conditioned information during fear extinction. Selective inhibition of the VTA^DA^‐OFC neurocircuit by opto/chemo‐genetic manipulation interrupted *S*‐Ketamine‐based PTSD treatment. OFC‐targeted TI‐NIBS could extend the therapeutic time window of *S*‐Ketamine, enabling the retention of efficacy if the optimum period for PTSD treatment is missed.

The use of ketamine to treat PTSD has been a major focus in recent clinical research. Several studies have shown its rapid and robust effects on PTSD symptoms, including intrusive memories, avoidance behaviors, hyperarousal, and heightened anxiety, fear extinction.^[^ ^7^, ^29^, ^30^, ^31^ ^]^ These symptomatic improvements can be found within hours and last up to 28 days, in contrast to the delayed effects of selective serotonin reuptake inhibitors (SSRI) such as paroxetine, sertraline, and fluoxetine, which can take weeks or months to achieve partial relief.^[^ ^29^, ^32^ ^]^ However, ketamine treatment for PTSD has some limitations. For instance, ketamine failed to reduce the long‐term prevalence of PTSD in trials involving military personnel and burn victims.^[^ ^33^ ^]^ Notably, the delivery time of ketamine‐assisted psychotherapy (KAP) before, during, or after psychotherapy has resulted in heterogeneous psychiatric outcomes.^[^ ^34^ ^]^ These phenomena suggest that drug delivery time might be crucial for the therapeutic efficacy of ketamine.

As a psychoactive substance with rapid emotional regulation, an unresolved issue remains: Does PTSD treatment with ketamine rely on early intervention to prevent the development of symptoms or direct behavioral modification at the onset of abnormal psychosis? To address this question, the dynamic change of the central nervous system throughout PTSD progression requires a clear illustration first. Dysfunction of midbrain VTA dopaminergic signaling contributes to PTSD symptoms. Furthermore, abnormal dopamine synthesis and transmission in brain regions such as the amygdala, hippocampus, and prefrontal cortex are associated with impaired fear memory processing, and restoring proper dopamine signaling can alleviate PTSD symptoms.^[^ ^35^, ^36^ ^]^ Altered VTA^DA^ neuronal activity is strongly implicated in PTSD and is characterized by a prolonged decrease in burst firing, which normally results in robust dopamine release.^[^ ^13^, ^37^ ^]^ In the current study, we demonstrated that PTSD modeling leads to the progressive suppression of neuronal activity, with a gradual decline in both overall firing rates and burst firing rates. This sustained decrease in VTA^DA^ neuronal excitability is recognized as a crucial factor in PTSD development following stress.

The excitability and firing patterns of VTA^DA^ neurons are determined by various receptors, ion channels, and related neurocircuitry. For example, HCN, SK2, and NMDA receptors mediate the disruption of intrinsic regulators of neuronal activity after stress.^[^ ^16^, ^38^ ^]^ Kv4.3 and BKCa1.1, differentially affect the dynamics of dopamine neuron firing and dopamine release during reinforcement and extinction learning.^[^ ^39^ ^]^ Moreover, PTSD‐induced deficits in synaptic transmission and plasticity are closely related to NMDA receptor hypofunction,^[^ ^40^, ^41^ ^]^ by which NMDA receptor (NMDAR) plasticity represents a potential neural substrate for conditioned dopaminergic neuron burst responses to environmental stimuli.^[^ ^42^ ^]^ In addition, the lateral habenular, as an anti‐reward center, is activated after stress and inhibits VTA^DA^ neurons through long projections.^[^ ^43^, ^44^ ^]^ Besides, astrocytes can regulate dopamine transmission by modulating the extracellular tone of axonal neuromodulators, including GABA and adenosine.^[^ ^45^ ^]^

In the present study, PTSD‐like behavior significantly improved when VTA^DA^ neuronal activity was repeatedly augmented through chemo‐genetic manipulation from the early stages of PTSD onset. Conversely, the chemo‐genetic inhibition of VTA^DA^ neurons attenuated the therapeutic effect of *S*‐Ketamine in ameliorating PTSD symptoms. Notably, we observed that the administration of *S*‐Ketamine only enhanced the excitability and burst firing of VTA^DA^ neurons when they were hyperactive (24 h after PTSD modeling). Recent evidence has shown that the function of ketamine is activity‐dependent and requires the target region to be intrinsically active with open NMDARs.^[^ ^43^ ^]^ Meanwhile, the prolonged modulatory effect of ketamine on neuronal excitability is potentially due to the use‐dependent trapping of ketamine in NMDARs, which keeps the receptors active longer, or through the enhancement of synaptic plasticity and neural circuit functioning.^[^ ^44^, ^46^ ^]^ The RNA sequencing and qRT‐PCR data also reveal that a transient upregulation of the expression of the majority of NMDA receptor (NMDAR) and AMPA receptor (AMPAR) subunits on day 1 post‐traumatic injury, followed by a return to baseline levels on day 7. Notably, GluN2B, a pivotal mediator of synaptic plasticity and long‐term potentiation (LTP), aligns this temporal pattern. Conversely, the loss of NMDA receptor plasticity (e.g., GluN2B downregulation) at 7 days post‐trauma potentially explains the diminished therapeutic efficacy of delayed *S*‐Ketamine administration. These findings provide potential molecular evidence linking PTSD progression to impaired VTA^DA^ neuron plasticity. Indeed, despite the action mechanisms of ketamine beyond NMDARs (e.g., GABAergic and dopaminergic modulation), our findings highlight NMDARs and AMPARs dynamics as a critical factor in its temporal therapeutic window. Further investigation is needed to dissect how synaptic mechanisms, including but not limited to NMDARs and AMPARs, collectively drive anti‐PTSD effects of *S*‐Ketamine.

Furthermore, VTA^DA^ neurons were sensitive to renewing conditioned stimuli during fear extinction. This variation was suppressed in PTSD mice and can be reversed by early *S*‐Ketamine intervention. Opto‐activation of VTA^DA^ neurons or VTA^DA^‐OFC circuits during aversive stimuli can facilitate fear extinction. Although earlier studies have predominantly focused on the medial prefrontal cortex (mPFC), emerging evidence highlights the distinct contributions of the OFC. Activation of top‐down projections from the OFC to the insular cortex enhances fear extinction,^[^ ^18^ ^]^ whereas the inhibition of OFC^CaMKII^ neurons significantly impairs fear extinction.^[^ ^19^ ^]^ Furthermore, the OFC forms extensive synaptic connections with important fear‐encoding regions, including the basolateral amygdala (BLA) and hippocampus. Mechanistically, OFC‐mediated inhibition of fear memory‐encoding neurons in the amygdala and hippocampus supports extinction memory retrieval.^[^ ^47^, ^48^ ^]^ Meanwhile, the OFC‐hippocampal pathway mediates antidepressant‐like effects following repetitive transcranial magnetic stimulation (rTMS).^[^ ^21^ ^]^ Therefore, we speculated that the VTA^DA^‐OFC circuit may mediate the replacement of conditioned information by eliminating aversive memory to restrict PTSD development.

Considering the functional specificity and accessibility in the clinic, we developed a TI‐NIBS technique. Through the application of two high‐frequency scalp currents with a slight frequency difference, TI‐NIBS generates a low‐frequency envelope waveform within the brain (20 Hz), enabling targeted stimulation of the OFC without disrupting the neural activity in other cortical areas.^[^ ^49^, ^50^ ^]^ This approach significantly enhances dopamine release in the OFC and sensitizes neuronal response to *S*‐Ketamine. Particularly, OFC‐targeted TI‐NIBS extended the therapeutic time window of *S*‐Ketamine.

This study had some limitations that warrant consideration. The marked receptor affinity differences between *S*‐Ketamine and racemic ketamine, combined with potential dissociative effects at the 10 mg kg^−1^ dose,^[^ ^51^ ^]^ highlight the need to evaluate lower‐dose regimens. Furthermore, although early *S*‐Ketamine intervention (24 h post‐trauma) ameliorated PTSD‐like behaviors in both sexes and revealed a conserved therapeutic time window (with delayed treatment at 7 days post‐modeling showing no efficacy) (Figure S11, Supporting Information), the SPS&S model lacks preclinical validation in females.^[^ ^52^ ^]^ Despite the observed sex‐divergent PTSD phenotypes and stress pathophysiology,^[^ ^53^ ^]^ the therapeutic efficacy and neurobehavioral mechanisms of ketamine in female patients with PTSD remain underexplored, necessitating a systematic investigation of sex‐specific responses. Besides, the conclusions were based solely on the SPS&S model. Incorporating additional PTSD paradigms would improve the generalizability of the findings.

## 4 Conclusion

Our study demonstrates that early rather than late administration of *S*‐Ketamine significantly alleviates PTSD‐like behaviors in mice, which is attributed to the reversal of the progressive suppression of VTA^DA^ neuronal activity. In addition, TI‐NIBS neuromodulation provides an optimal and convenient clinical strategy for ketamine‐based PTSD treatment.

## 5 Experimental Section

### Mice

The animal experiments were approved by the Ethics Committee for Animal Experimentation and performed in strict compliance with the guidelines established by the Fourth Military Medical University (Xi'an, China) and the ARRIVE (Animal Research: Reporting of In Vivo Experiments) guidelines. Mice were procured from the Animal Center of the Fourth Military Medical University. All experiments were performed on adult male mice aged 8–12 weeks. Mice were housed in a controlled environment with a temperature range of 22–24 °C and relative humidity maintained at 50%–60%, without any restricted access to food and water.

### Drug

*S*‐Ketamine hydrochloride was provided by Jiangsu Hengri Pharmaceutical Co., Ltd. (Lianyungang, China) and diluted in 0.9% saline. Saline (0.9%) was used as the vehicle control.

### PTSD Modeling (SPS&S Stress)

Based on previous methodologies,^[^ ^22^ ^]^ the mice were exposed to four distinct stressors in a sequence: 4 h of restraint stress, 30 min of forced swimming (25 °C), deep anesthesia with ether, and an unconditioned foot shock (0.8 mA for 5 s). After each stressor, the mice were allowed to rest in their home cages for 30 min before exposure to the next stressor.

### Fear Conditioning and Extinction

All auditory fear conditioning and extinction procedures were performed using the Ugo Basile Fear Conditioning System (UGO BASILE SRL, Italy). The mice were habituated to the conditioning (context A) and extinction chambers (context B) over two consecutive days. During the fear conditioning phase, the mice were placed in the conditioning chamber (context A) and allowed to acclimate for 180 s. Following this, they received five pairings of the conditioned (CS, 78 dB, 4.5 kHz, 30 s each) and unconditioned stimulus (US, 0.6 mA, 2 s each), the CS was delivered at variable intervals ranging from 60 to 180 s. Each US was immediately followed by a 2‐s foot shock (US, 0.6 mA). Sixty seconds after the final tone, the conditioned mice were returned to their home cages. After each trial, the cages were cleaned with 75% ethanol.

Regarding fear extinction, the mice that underwent conditioning in context A were placed in the extinction chamber (context B) for the next 2 days. During this period, they were exposed to 12 CS presentations each day (78 dB, 4.5 kHz, 30 s each, with inter‐tone intervals of 60–90s) but without the paired foot shock. The chambers were cleaned with a 1% peppermint‐scented solution between animals.

Extinction retrieval was conducted 24 h after the final extinction session, where the mice underwent 12 additional CS presentations (same parameters as before) in context B. Two hours after extinction retrieval, the mice were returned to context A for a 5 min session to assess fear renewal.

Mouse movement in contexts A and B was monitored, and the data were analyzed using ANY‐maze software. Fear response was defined as a behavioral freeze lasting for more than 1s. The freezing time ratio for each CS presentation was also calculated. Animals for fear extinction test were excluded if they exhibited a freezing time ratio of less than 20% during the final CS presentation in the training stage of fear conditioning training, indicating insufficient fear memory acquisition. Animals were excluded if they exhibited 1) a freezing time ratio ≤ 20% during the final CS presentation in the training stage of fear conditioning, indicating insufficient fear memory acquisition; or 2) a freezing time ratio ≥90% before the first CS presentation in the training stage of fear conditioning training, which reflects impaired baseline mobility.

### 3D Motion‐Capture and Spontaneous Behavior Decomposition

To quantitatively assess spontaneous rodent behavior, we employed 3D motion‐capture and behavioral decomposition techniques, as previously described by Huang et al.^[^ ^23^ ^]^ in the hierarchical 3D‐motion learning framework. The pipeline comprises three main steps (Figure S12, Supporting Information): synchronized multi‐view video collection, 3D pose reconstruction, and subsequent decomposition of behavioral dynamics from skeletal trajectories. These procedures were implemented using a 3D‐AI Mouse Behavior Analysis System (BayONE Scientific, Guangdong, China; https://bayonesci.com/behavioratlas/), which integrates both hardware and software components for automated multi‐camera synchronization, robust 3D skeleton tracking, and machine learning‐based behavior parsing. The cameras placed at each of the four corners of the apparatus simultaneously recorded the spontaneous behavior of the mice for 15 min. The behavior fraction was calculated by dividing the duration of a specific behavior by the total duration of all the behaviors exhibited. Methodological details are described in the Experimental Section (Supporting Information).

### In Vivo Electrophysiology

For in vivo single‐unit recordings, 16‐channel tetrode microelectrodes (Kedou BC Co., Ltd., Suzhou, China) were implanted into the unilateral VTA of *DAT‐cre* mice. To isolate neuronal spikes, the recorded signals were sampled at 30 kHz and processed using a bandpass filter with a frequency range of 250–5000 Hz. Spontaneous neural firing was recorded at four time points: one day prior to PTSD modeling, one day after induction, 7 days after modeling, and 8 days after modeling. Each recording session lasted 30 min. To assess the immediate effect of *S*‐Ketamine, a 30 min baseline recording was conducted, followed by a 30 min recording. Single‐unit activity was sorted using a threshold and shape detection method based on principal component analysis (PCA) with Offline Sorter software (KlustaKwik, USA). Spikes with inter‐spike intervals <2 ms were excluded from further analysis. Cross‐correlation histograms were used to remove cross‐channel artifacts. In addition, units with a baseline firing rate below 0.1 Hz were excluded.

To identify VTA^DA^ neurons, a 473 nm laser with a frequency of 20 Hz and a pulse width of 20 ms was applied for 10 s during the first 15 min of recording. Neurons were classified as expressing AAV2/9‐Ef1a‐DIO‐ChR2‐mCherry if the spikes were reliably evoked by laser pulses (with a reliability score >0.7 for all units), had a short first‐spike latency (<4 ms for all units), and displayed minimal jitter (<3 ms for all units). In addition, cross‐correlation analysis (MATLAB xcorr function) was used to quantitatively validate the waveforms of laser‐evoked spikes against spontaneous spikes. This approach computes correlation sequences across all time lags, identifies maximum absolute correlations, and normalizes by the product of the waveforms' L2‐norms, yielding a near‐perfect Pearson correlation coefficient between conditions. The waveforms of the laser‐evoked spikes were highly similar to those of the spontaneous spikes.

Data analysis was performed using NeuroExplorer5 (Plexon Inc.) and MATLAB. A burst event was defined as the occurrence of at least two spikes within 80 ms or less, followed by a silent period of at least 160 ms. The first 15 min of each recording and the 200 s immediately following drug administration were excluded from the analysis. To quantify burst activity, a burst index was calculated as the ratio of the number of spikes within bursts to the total number of spikes recorded.

### Fiberphotometry Recording

Fiberphotometry recording (ThinkerTech, Nanjing, China) enables continuous monitoring of neuronal activity or extracellular release of neurotransmitters by measuring fluorescence signals.^[^ ^54^ ^]^ The laser intensity at the fiber tip was consistently maintained between 30 and 40 µW. To normalize the fluorescence signals, the ΔF/F ratio was computed as (F–F_0_) / F_0_, where F_0_ represents the baseline fluorescence, and F denotes the real‐time fluorescence signal.

For calcium imaging, AAV‐DIO‐GCaMP6s was injected into the VTA of DAT‐cre mice with implanted optical fibers (200 µm in diameter, 0.37 NA). Fluorescence intensities were recorded during fear extinction. The pre‐CS period, defined as 5s before the onset of CS, served as the baseline. The data were segmented according to specific behavioral events in individual trials. The peak ΔF/F was calculated as the maximum fluorescence variation observed during the CS presentation.

To monitor DA levels in the OFC, AAV‐hSyn‐DA3m cells were transfected. For VTA^DA^ neuronal activation, the baseline was set to 2 s preceding activation onset. During TI stimulation, the baseline was defined as 200 s prior to injection onset, with data from 120 s after administration excluded from the analysis. The peak ΔF/F was determined as the maximal fluorescence change observed within 5 s following activation of VTA^DA^ neurons or within 20 min post *S*‐Ketamine administration.

### Chemogenetic Manipulation After PTSD Modeling

AAV‐DIO‐hM3Dq‐mCherry, AAV‐DIO‐hM4Di‐mCherry, or AAV‐DIO‐mCherry was bilaterally injected into the VTA of DAT‐Cre mice. After allowing 3 weeks for viral expression, CNO 1 mm) was administered via intraperitoneal injection on the first, third, fifth, and seventh day after PTSD modeling. In the experiment of inhibiting VTA^DA^ neurons, *S*‐Ketamine was administered on the first day.

### Opto‐Genetic Manipulation During Fear Extinction Training

For opto‐excitation or inhibition of DA neurons during behavioral assays, the procedure followed a previously described method.^[^ ^18^ ^]^ Blue light (473 nm, 4–6 mW) was delivered in 20 Hz bursts of 20 ms pulses lasting 250 ms, occurring every 5 s during the presentation of every 30 s CS. Yellow light (594 nm) was delivered in a continuous pattern every 30 s of CS. The output power was 8–10 mW.

### Temporally Interfering Non‐Invasive Brain Stimulation (TI‐NIBS)

Based on a previous study,^[^ ^49^ ^]^ a mouse head model was created using CT and MRI data from realistic mouse heads. The mouse cranial vault was divided into more than 100 regions as potential electrode embedding sites. The finite element method and optimization framework were used to solve the multi‐objective optimization problem and obtain the optimal cranial‐parietal electrode sites corresponding to the target stimulated brain regions. The first pair was implanted at AP: 3 mm, ML: +2.7 mm, and AP: 3 mm, ML: −1 mm. The second pair was positioned at AP: 3 mm, ML: −2.7 mm, and AP: −3 mm, ML: 3.7 mm. These placements were guided by the standard brain atlas coordinates to ensure precise targeting of the regions for TI‐NIBS. Two high‐frequency alternating currents (2000 and 2020 Hz, 150 µA) were applied to the OFC region via scalp electrodes, generating an envelope wave at 20 Hz (Δf = 20 Hz), which selectively modulated deep cortical neurons without affecting the surrounding tissue. After *S*‐Ketamine was injected intraperitoneally, TI‐NIBS was administered immediately for 20 min on the 7th day after PTSD modeling.

### Statistics

Data collection and processing were performed randomly to reduce potential bias. Behavioral experiments and data analyses were performed under blinded conditions. Statistical analyses were performed using GraphPad Prism 10. The Shapiro–Wilk test was used to evaluate data normality, and variance homogeneity among groups was assessed. For data with a normal distribution and equal variances, statistical comparisons were performed using paired or unpaired *t*‐tests and analysis of variance (ANOVA), followed by Tukey's post‐hoc test. Non‐normally distributed data were analyzed using the *Friedman* test. All statistical details are presented in Table S2 (Supporting Information). Data are presented as means ± SEM, and the statistical thresholds for significance were defined as ^*^ *p* <0.05, ^**^ *p* <0.01, ^***^ *p* <0.001, and ^****^ *p* <0.0001.

## Conflict of Interest

The authors declared no conflict of interest.

## Supporting information



Supporting Information

Supplemental Table 2

## Data Availability

All data required to evaluate the conclusions of this study are presented in the paper and/or Supplementary Information.
