# Supplementary material for: Time‐Dependent Therapeutic Effect of S‐Ketamine on PTSD Mediated by VTA‐OFC Dopaminergic Neurocircuit
Source: Adv Sci (Weinh). 2025 Sep 25;12(46):e00805. doi: 10.1002/advs.202500805 (PMC12697765; doi:10.1002/advs.202500805)
Supplement: Supplementary file 1 — Supporting Information [file ADVS-12-e00805-s002.docx]

**Supplementary Materials**

**Time-dependent therapeutic effect of *S*-ketamine on PTSD mediated by VTA-OFC dopaminergic neurocircuit**

By *Ye Wang* et al.

**Supplementary Experimental Section**

***3D behavioral analysis*:** 3D Motion Data Collection: Four synchronized Intel RealSense D435 RGB-D cameras (940 × 540 resolution, 30 FPS) were orthogonally mounted on a device cabin to ensure 360° spatial coverage. A white acrylic arena (diameter: 38 cm) with uniform LED illumination minimized environmental noise. Behavioral recordings spanned 15 minutes per subject.

3D Pose Reconstruction: 2D keypoints of 16 anatomical landmarks (nose, ears, limbs, tail) were extracted from each camera view using a pre-trained DeepLabCut model. Multi-view geometry constraints (triangulation) fused 2D coordinates into 3D skeletal trajectories, achieving sub-millimeter accuracy validated via manual tracking of 2500 frames across 10 mice.

Behavioral Decomposition and Unsupervised Clustering: We developed a two-stage decomposition strategy to extract spontaneous behavioral modules from 3D skeletal data. First, the animal’s posture trajectories were temporally reduced to representative poses through center alignment and redundancy filtering. Then, adjacent postural segments were grouped into non-locomotor movement (NM) units using a model-free similarity metric - Dynamic Time Alignment Kernel (DTAK) - which accounts for temporal variability across sequences.

A pairwise similarity matrix was constructed and optimized via dynamic programming, allowing robust segmentation without prior labels (Fig.S11). These NM modules were further embedded into a low-dimensional space using UMAP, and combined with velocity-based locomotion features. Final behavioral phenotypes were identified through hierarchical clustering, yielding a structured map of spontaneous movements for downstream phenotyping and group-level comparisons. Following this, supervised classification was used to identify distinct clusters of behavioral movements according to DSM-V guideline of PTSD. The behavior fraction was calculated by dividing the duration of a specific behavior by the total duration of all behaviors exhibited.

***Viral injections:*** Mice were anesthetized using a 1% sodium pentobarbital solution (10 ml/kg, i.p.). Afterward, each animal was secured in a stereotactic frame equipped with non-rupture ear bars (RWD Life Science, Shenzhen, China). A midline incision was made in the scalp, followed by the creation of small bilateral craniotomies using a microdrill with 0.5 mm burrs. And the viruses were injected using a microinjection pump combined with a glass microtubule (tip diameter: 10 - 20 μm). And the virus was injected 300 nl on each side, at a rate of 0.1 μl/min. To allow the virus to diffuse adequately, the syringe is left in place for an additional 10 min after each injection (AP, anteroposterior to bregma; ML, lateral to the midline; DV, below bregma; all in mm).

For fiber photometry and in vivo electrophysiology experiments, virus was unilaterally injected into VTA or OFC. VTA: AP, -3.3; ML, +0.45; DV, -4.25; angle 0°; OFC: AP, +2.15; ML, +0.75; DV, -2.8; angle 0°. For opto/chemo-genetic manipulation, virus was injected bilaterally, VTA: AP, -3.3; ML, +0.45; DV, -4.25; angle 0°; VTA: AP, -3.3; ML, -1.16; DV, -4.31; angle 10°; OFC: AP, +2.15; ML, +0.75; DV, -2.8; angle 0°; OFC: AP, +2.15; ML, -1.24; DV, -2.84; angle 10°. And for optogenetic manipulation optical fiber was implanted at the same coordinates simultaneously.

Following the procedure, the mice were allowed a recovery period of at least three weeks before behavioral testing and other experiment. The injection sites were subsequently verified by the expression of fluorescent proteins, such as EGFP or mCherry, at the conclusion of the experiment.

AAV2/9-Ef1α-DIO-ChR2-mCherry, AAV2/9-Ef1α-DIO-eNpHR3.0-mCherry, AAV2/9-Ef1α-DIO-hM4Di-mCherry, AAV2/9-Ef1α-DIO-hM3Dq-mCherry, AAV2/9-Ef1α-DIO-mCherry, AAV2/9-Ef1α-DIO-GCaMp6s were purchased from Brain-VTA Co., Ltd (Wuhan, China). AAV2/9-hSyn-DA3m was purchased from WZ Biosciences Co., Ltd (Shandong, China).

***Open field test (OFT):*** Mice were initially placed in the center of the open field chamber (40 cm ⅹ 40 cm ⅹ 30 cm). The movement trajectory of the mice was recorded over a 5 min using a camera placed above the open field. The field was divided into a central zone (20 cm × 20 cm) and a peripheral zone for analysis. The total distance and the numbers of entries to central zone were analyzed using ANY-maze software. The apparatus was thoroughly cleaned with 75% ethanol after each session.

***Elevated plus maze (EPM) test:*** The elevated plus maze apparatus (Global Biotech Inc., Shanghai) was made of opaque materials and included a central platform (10 cm × 10 cm), two open arms (50 cm × 10 cm), and two enclosed arms (50 cm × 10 cm) with protective walls 40 cm high, positioned 70 cm above the ground. At the start of each trial, animals were placed on the central platform facing one of the open arms and allowed to explore the maze for 5 min. Mouse movement was recorded using an overhead camera. And times in open zone and numbers of entries to open zone were analyzed using ANY-maze software. The apparatus was cleaned with 75% ethanol before and after each session.

***In vitro Electrophysiology:*** Mice were first anesthetized with isoflurane and then intracardially perfused with an ice-cold cutting solution, which contained 92 mM NMDG, 2.5 mM KCl, 1.25 mM NaH_2_PO_4_, 30 mM NaHCO_3_, 20 mM HEPES, 25 mM glucose, 2 mM thiourea, 5 mM Na-ascorbate, 3 mM Na-pyruvate, 0.5 mM CaCl_2_, and 10 mM MgSO_4_. Coronal brain slices (300 μm thick) including VTA were prepared in this solution, which was continuously bubbled with 95% O_2_ and 5% CO_2_, using a VT1200S Vibratome (Leica Microsystems). Following cutting, the slices were transferred to an interface chamber containing artificial cerebrospinal fluid (ACSF) with the following composition: 124 mM NaCl, 2.5 mM KCl, 1.25 mM NaH_2_PO_4_, 24 mM NaHCO_3_, 12.5 mM glucose, 5 mM HEPES, 2 mM CaCl_2_, and 2 mM MgSO_4_, and were bubbled with carbogen at 34°C for 45 min. After recovery, the slices were kept at room temperature until the recordings began.

Subsequently, slices were placed in a recording chamber and continuously perfused with ACSF at a flow rate of 1 - 3 ml/min. Visualizations were performed using an upright fixed-stage microscope (Olympus) equipped with both epifluorescence and infrared differential interference contrast (DIC) illumination to identify fluorescently tagged neurons and to conduct optogenetic stimulation. Patch-clamp electrodes (3-6 MΩ resistance) were pulled from borosilicate glass and filled with a potassium-based intracellular solution containing 145 mM K-Gluconate, 10 mM HEPES, 1 mM EGTA, 2 mM Mg-ATP, 0.3 mM Na_2_-GTP, and 2 mM MgCl_2_.

Whole-cell patch-clamp recordings were conducted in current-clamp mode at intrinsic resting membrane potentials to measure action potentials (APs). For optogenetic stimulation, neurons expressing ChR2 were activated with 473 nm blue light (delivered at frequencies ranging from 5-30 Hz with a 20 ms pulse duration), while neurons expressing eNpHR3.0 were inhibited using 594 nm yellow light (delivered at 1 Hz for 1 s). Light was delivered through an optical fiber coupled to an LED light source positioned above the recorded cell. Data were collected and processed using an Axon 700B Amplifier (Molecular Devices) and pClamp 10 software (Molecular Devices, version 10.6).

***RNA sequencing:*** After euthanasia by KCl intracardiac injection, the ventral tegmental area (VTA) was rapidly dissected from mouse brains and homogenized using a 1 mm diameter punch. Total RNA was extracted using the Total RNA Extractor (Trizol) kit (B511311, Sangon, China) following the manufacturer’s protocol, followed by DNase I treatment to eliminate genomic DNA contamination. RNA integrity was verified via 1.0% agarose gel electrophoresis, and quantification was performed using a NanoPhotometer® spectrophotometer (IMPLEN, CA, USA) and Qubit® 2.0 Fluorometer (Invitrogen). Polyadenylated mRNA was enriched from total RNA using poly-T oligo-attached magnetic beads. Sequencing libraries were constructed with the VAHTSTM mRNA-seq V2 Library Prep Kit for Illumina® according to the manufacturer’s instructions, including fragmentation by divalent cations, cDNA synthesis with M-MuLV Reverse Transcriptase, and adapter ligation. Libraries were size-selected (150 - 200 bp) using the AMPure XP system (Beckman Coulter, Beverly, USA) and sequenced on the NovaSeq sequencers (Illumina, San Diego, CA) to generate paired-end reads, achieving approximately 30 million reads per sample.

All animal groups (Sham, Post Day1, Post Day7) included five biological replicates. RNA purification, library preparation, and sequencing were performed by Sangon Biotech (Shanghai, China). Raw sequencing data underwent quality control using FastQC (version 0.11.2) and Trimmomatic (version 0.36) to remove adapters, low-quality bases (Q < 20), and short reads (< 35 nt), ensuring clean data for downstream analysis.

***qPCR assay:*** To determine gene expression in VTA between the different groups, an RT-qPCR test was conducted. Total RNA was isolated from tissues, and cDNA synthesis was performed using Maxima Reverse Transcriptase (Thermo Scientific) with random hexamer primers and dNTP Mix under the following conditions: 25 °C for 10 min, 50 °C for 30 min, and 85°C for 5 min. Amplification was carried out on a LightCycler480 II Real-Time PCR System (Roche, Rotkreuz, Switzerland) with 2 × SGExcel FastSYBR Mixture (Sangon Biotech, Shanghai, China). Primer pairs were designed using Primer Premier 5.0 software following specific criteria: GC content 20 - 80%, Tm 55 - 60 °C, avoidance of secondary structures, and 3’ - end nucleotide restrictions. Primer sequences for target genes (e.g., GluN1, GluA1) and the reference gene (Β-actin) are listed below:

M-GluN1-F1, GTGCTGTTATGGCTTCTGCG;

M-GluN1-R1, TTTGTTGCTGTTGTTTACCCG;

M-GluN2A-F4, GCTACACACTCTGCACCAATTTAT;

M-GluN2A-R4, TTCTCCCACTTGCCCACC;

M-GluN2B-F3, CATCTGAGCATCGTTACCTTGG;

M-GluN2B-R3, TGTAGCCTGGTTCCTCATCTGTT;

M-GluN2C-F1, TCCACGGCATCGTCTTTGA;

M-GluN2C-R1, CGCTCCAGTCGTATTCCTCC;

M-GluN2D-F3, CGCTGTGTGGGTGATGATGT;

M-GluN2D-R3, CACTGGCACGGAGTTGTTGA;

M-GluN3A-F3, CCTGAAGAATGATCCAGAGAAACT;

M-GluN3A-R3, GGGAGGGAGACCAATGCC;

M-GluN3B-F4, AACAGCACGCACCTTGGC;

M-GluN3B-R4, CCATACCTTGAAATGCCGAGA;

M-GluA1-F1, CTTCATCACTCCAAGTTTTCCC;

M-GluA1-R1, TCTTCTCGGCGGCTGTATC;

M-GluA2-F3, GAACGGCGTGTAATCCTTGAC;

M-GluA2-R3, CACCAGGGAGTCGTCGTAGTC;

M-GluA3-F2, AACCAGAACACCACTGAGAAGC;

M-GluA3-R2, GAACTGGGAGCAGAAAGCATT;

M-GluA4-F2, TGGAGGAGCAAATGTCACTGG;

M-GluA4-R2, GCCATTACCAAGACACCATCG;

M-β-actin-F, GTGCTATGTTGCTCTAGACTTCG;

M-β-actin-R, ATGCCACAGGATTCCATACC;

The relative expression of the target genes was normalized to β-actin levels.

***Histology and fluorescent immunostaine:*** Animals were deeply anesthetized using a 1% sodium pentobarbital solution and underwent transcardiac perfusion. The perfusion procedure involved the administration of 50 mL of 0.9% saline, followed by 50 mL of 4% paraformaldehyde (PFA) in 0.2 M phosphate buffer (pH 7.4). Following perfusion, the brains were post-fixed in 4% PFA at 4 °C for 2 hours. After fixation, the brains were progressively dehydrated through a series of solutions, beginning with 30% sucrose in PBS.

The brains, containing VTA and OFC were coronally sectioned into 40 μm thick slices using a cryostat microtome (Leica CM3050, Germany). The sections were then washed three times for 10 min each in phosphate-buffered saline (PBS, pH 7.4) and subsequently blocked with 5% normal donkey serum (NDS) in PBS with 0.3% Triton X-100 (PBST) for 2 hours at room temperature. The primary antibodies, diluted in 2.5% NDS in PBST, were incubated 48 h at 4^o^C. Following primary antibody incubation, the sections were exposed to secondary antibodies, also in 2.5% NDS in PBST, for 2 hours at room temperature. After the incubation, the tissue sections were rinsed three times for 10 min each in PBS and then mounted for imaging. The brain slices were imaged using a slide scanner (VS200, Olympus, Japan) and a laser confocal fluorescence microscope (FV1200, Olympus, Japan). Quantification was carried out by counting the c-fos-positive cells in the brain sections. All cell counts were performed in a blinded manner with respect to treatment groups to ensure unbiased results.

The following antibodies were used for immunostaining: primary antibodies included guinea pig anti-c-fos (1:1000, 266308, Synaptic Systems, USA) and mouse anti-tyrosine hydroxylase (TH, 1:1000, 822301324, GeneTex, USA). Secondary antibodies included Alexa Fluor 488-conjugated anti-guinea pig (1:500, 706545148, Jackson ImmunoResearch, USA), Alexa Fluor 594-conjugated anti-mouse (1:800, 715585150, Jackson ImmunoResearch, USA), and Alexa Fluor 488-conjugated anti-mouse (1:800, 715545151, Jackson ImmunoResearch, USA).

***Uniform manifold approximation and projection (UMAP):*** We use UMAP to reduce high-dimensional data to low-dimensionality, details are as follows:

The core formulas:

$$w_{ij}=exp\left( -\frac{max(0,d(x_{i},x_{j})-\rho_{i}}{\sigma_{i}} \right)$$

$$q_{ij}=\left( 1+a\cdot y_{ij}^{2b} \right)^{-1}$$

$$L = \sum_{i<j} w_{ij}log\left( \frac{w_{ij}}{q_{ij}} \right)+\left( 1-w_{ij} \right)log\left( \frac{1-w_{ij}}{1-q_{ij}} \right)$$

The core parameters:

'n_neighbors', controlling the number of neighbors to consider when constructing a high-dimensional graph, which determines how the algorithm balances the local and global structure, we set it to 30; 'min_dist', to set the minimum spacing between points in a low-dimensional space, we choose the default 0.1; 'metric', to represent the distance calculation method for high-dimensional spaces, we have chosen the default Euclidean geometric distance; 'n_components', representing the dimensionality reduction target dimension, we set it to 2.

We use Matlab2024a to perform the calculations, and the code is as follows:

“umap_params = struct ('n_neighbors',30,'min_dist',0.1, ...

'metric','euclidean','n_components',2);

reducer = UMAP (umap_params);

embedding = reducer.fit_transform (data);”

**Supplementary** **Figures and Figure Legends**


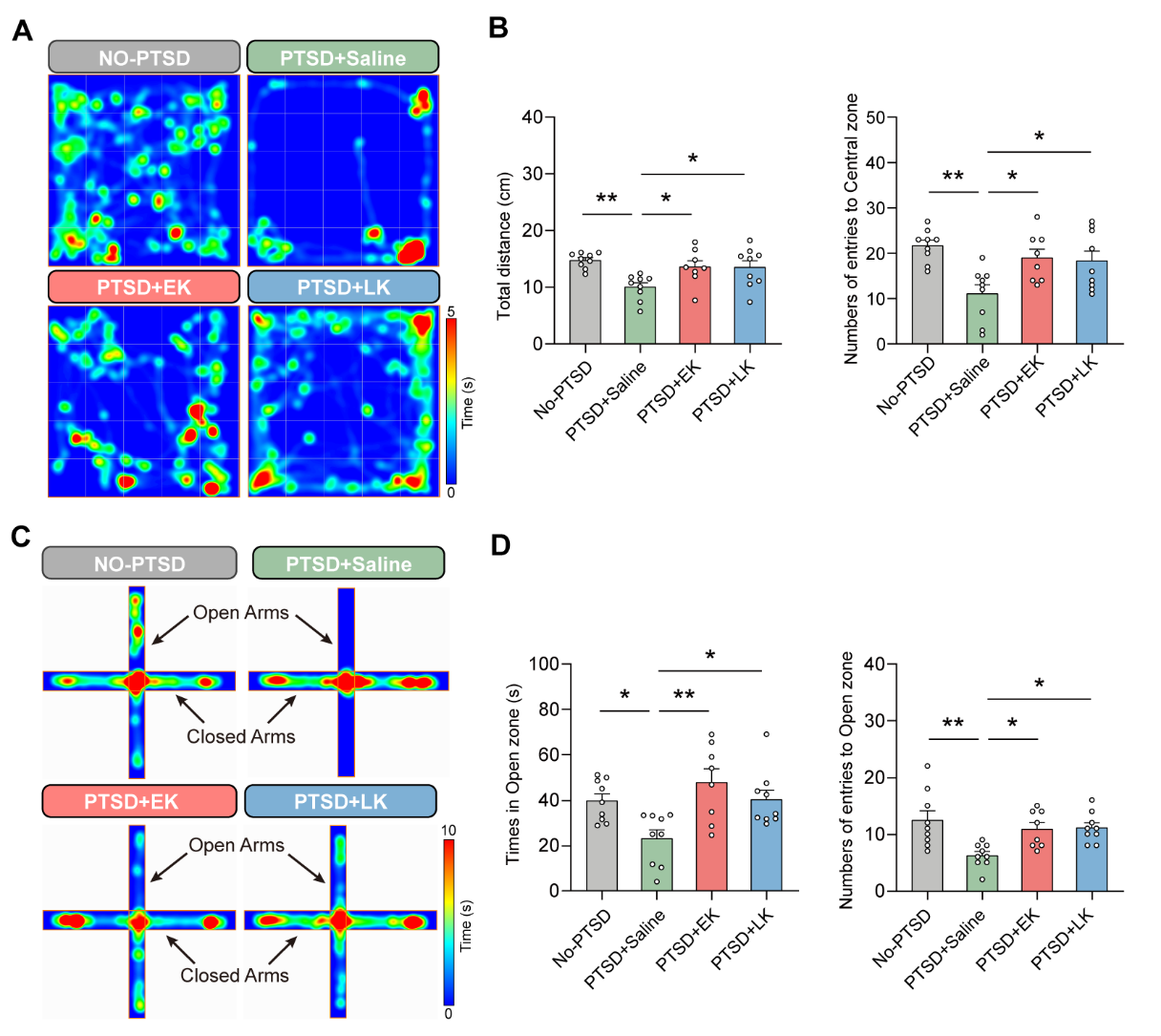


**Fig.S1. Both early and late administration of *S*-Ketamine alleviate anxiety behaviors induced by PTSD. (A)** Representative heat maps during OFT. (**B**) Total distance traveled (left, F (3, 31) = 1.377, *P* = 0.0046), numbers of entries to center zone (right, F (3, 31) = 1.073, *P* = 0.002) in OFT. **(C)** Representative heat maps during EPM. **(D)** times in open zone (left, F (3, 31) = 1.094, *P* = 0.0027) and numbers of entries to open zone (right, F (3, 31) = 1.815, *P* = 0.0032) in EPM. NO-PTSD, PTSD+Saline, PTSD+LK, *n* = 9 mice, PTSD+EK, *n* = 8 mice. Data are presented as means ± SEM, **P* < 0.05, ***P* < 0.01, One-way ANOVA with *Tukey* test.


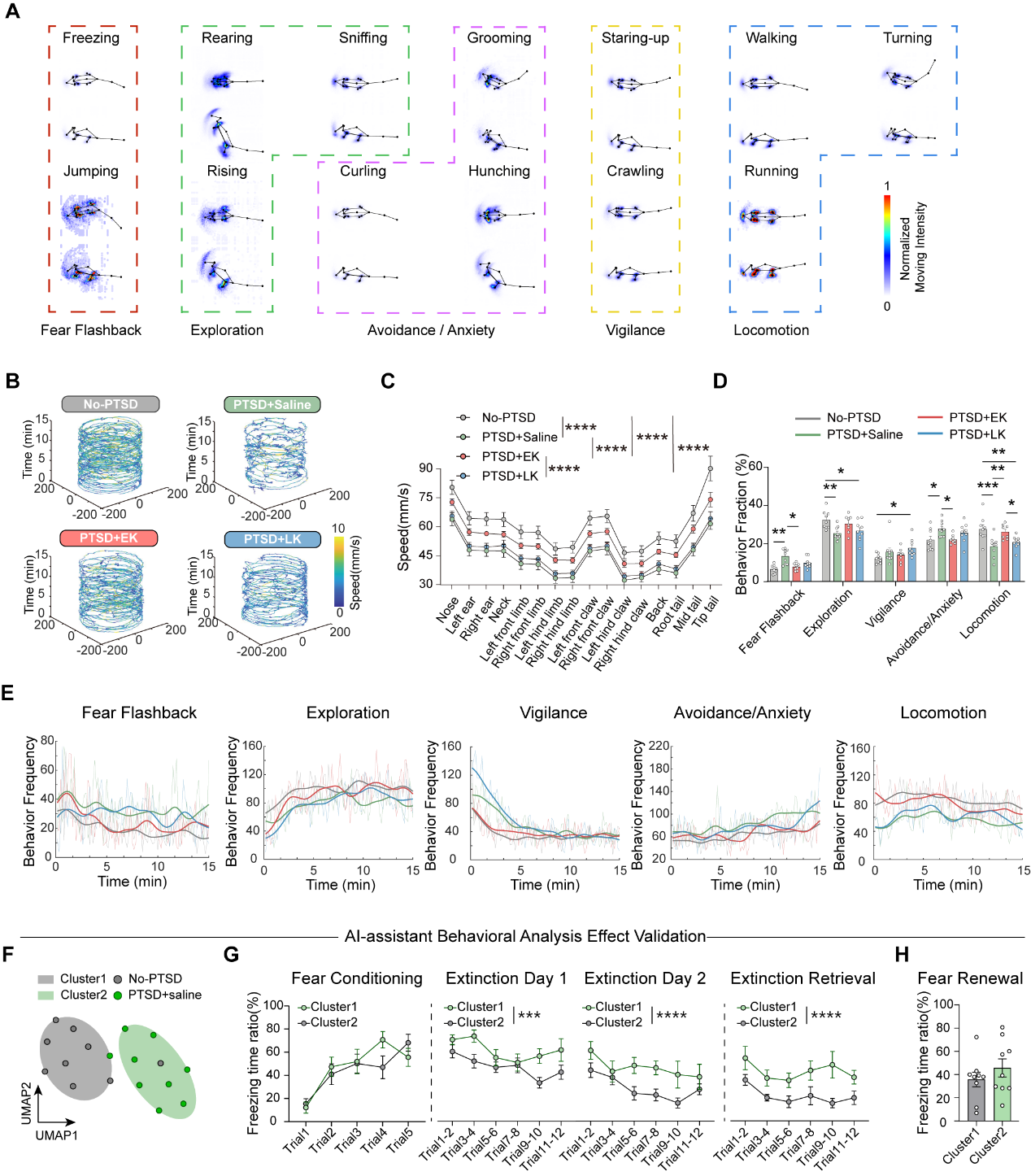


**Fig.S2. Early administration of *S*-ketamine mitigates PTSD-like behavior.** (**A**) Average skeleton positions from all frames within each movement phenotype. (**B**) Representative of speed dynamics. (**C**) The speed of 16 body points across four groups of mice. F (3, 512) = 129.9, *P* < 0.0001, two-way ANOVA. (**D**) Frequency of five behavioral categories among four groups. (**E**) Faction of five behavioral categories, F (3, 155) = 4.809e-011, *P* > 0.9999, two-way repeated measures ANOVA. No-PTSD, PTSD+Saline, PTSD+LK, *n* = 9 mice, PTSD+EK, *n* = 8 mice. (**F**) All samples were clustered into two clusters according to their 3D spontaneous behavior, and No-PTSD accounted for 88.9% in cluster 1, 88.9% of PTSD saline in cluster 2. (**G**) Freezing responses to the CS during fear conditioning (F (1, 80) = 0.4073, *P* = 0.5251), extinction (Day 1: F (1, 96) = 15.33, *P* = 0.0002; Day 2: F (1, 96) = 16.93, *P* < 0.0001) and extinction retrieval (F (1, 96) = 27.32, *P* < 0.0001), two-way repeated measures ANOVA. (**C**) Freezing responses during fear renewal, t (16) = 0.9958, *P* = 0.3342, unpaired *t* test. Data are presented as means ± SEM, **P* < 0.05, ***P* < 0.01, ****P* < 0.001, *****P* < 0.0001.


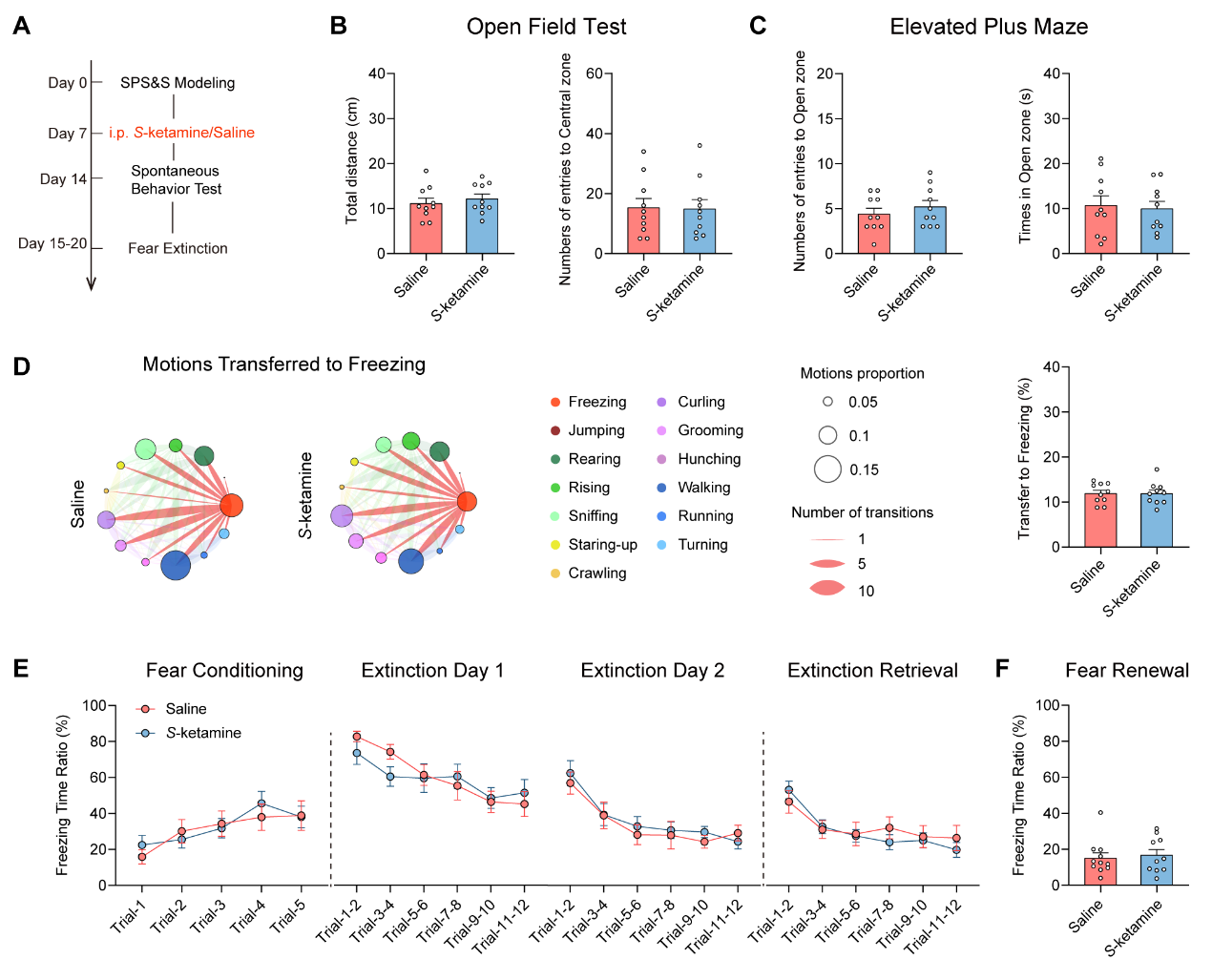


**Fig.S3. Inefficacy of late *S*-ketamine intervention is independent on the duration of drug delivery.** (**A**) Schematic of drug injections and behavioral tests. (**B**) Behavioral performance in open field test. Total distance traveled (left, t (18) = 0.6604, *P* = 0.5173), numbers of entries to center zone (right, t (18) = 0.09169, *P* = 0.928), unpaired *t* test. (**C**) Behavioral performance in elevated plus maze test. Numbers of entries to open zone (left, t (18) = 0.8485, *P* = 0.4073) and times in open zone (right, t (18) = 0.2725, *P* = 0.7884), unpaired *t* test. (**D**) Movements transferred to freezing. The size of the colored circle represents the proportion of the corresponding behavior, and the diameter of the line segment represents the number of transformations. T (18) = 0.01323, *P* = 0.9896, unpaired *t* test. (**E**) Freezing responses to the CS during fear conditioning (F (1, 95) = 0.1029, *P* = 0.749), extinction (Day 1: F (1, 108) = 0.2066, *P* = 0.6504; Day 2: F (1, 108) = 0.5186, *P* = 0.473) and extinction retrieval (F (1, 108) = 0.2749, *P* = 0.6012), two-way repeated measures ANOVA. (**F**) Freezing responses during fear renewal, U = 52, *P* = 0.8633, Mann-Whitney *U* test. Data are presented as means ± SEM.


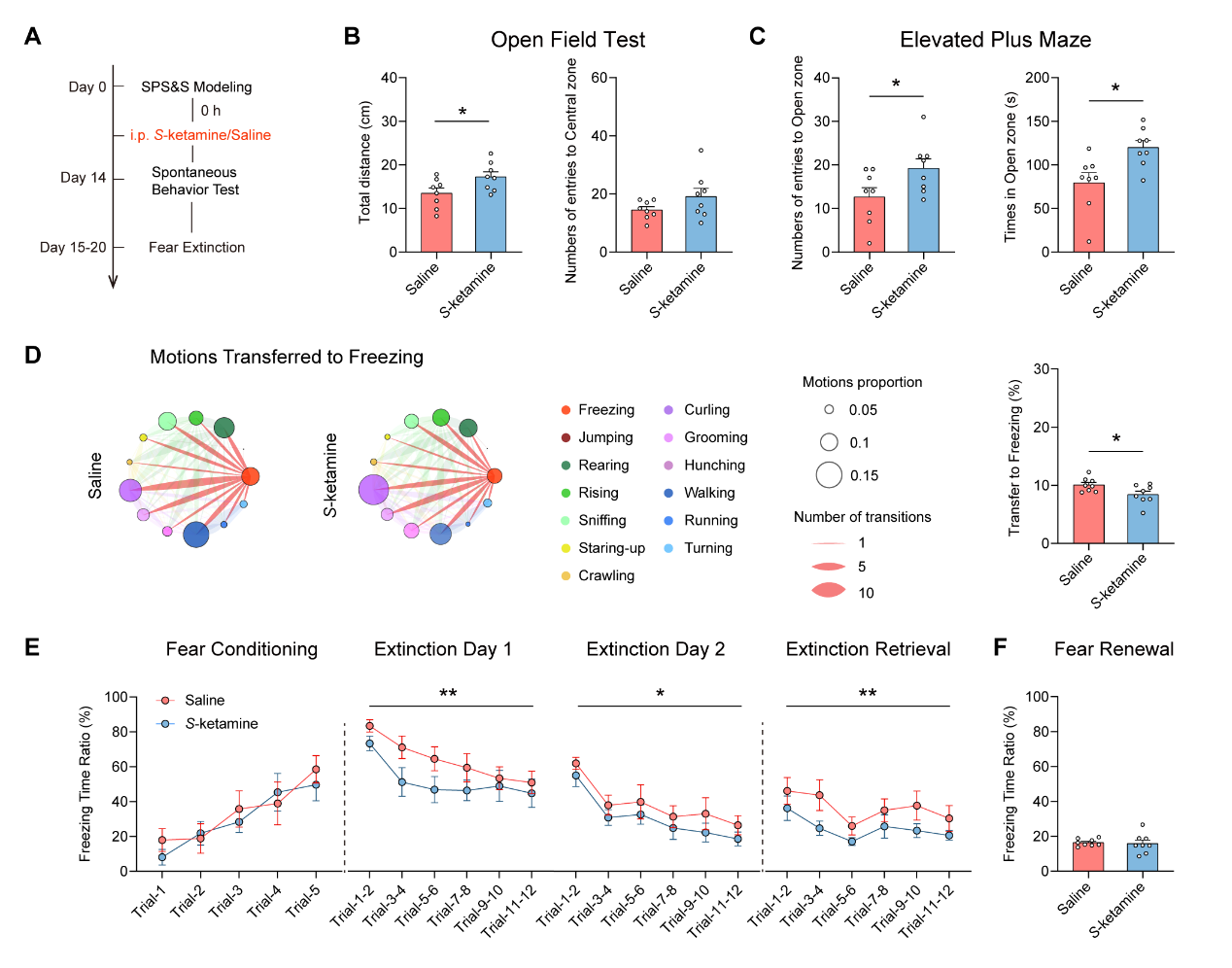


**Fig.S4. *S*-ketamine administration at 6h after PTSD modeling ameliorates PTSD-like behaviors.** (**A**) Schematic of drug injections and behavioral tests. (**B**) Behavioral performance in open field test. Total distance traveled (left, t (14) = 2.272, *P* = 0.0394), numbers of entries to center zone (right, t (14) = 1.537, *P* = 0.1465), unpaired *t* test. (**C**) Behavioral performance in elevated plus maze test. Numbers of entries to open zone (left, t (14) = 2.171, *P* = 0.0476) and times in open zone (right, t (14) = 2.913, *P* = 0.0113), unpaired *t* test. (**D**) Movements transferred to freezing. The size of the colored circle represents the proportion of the corresponding behavior, and the diameter of the line segment represents the number of transformations. t (14) = 2.267, *P* = 0.0397, unpaired *t* test. (**E**) Freezing responses to the CS during fear conditioning (F (1, 70) = 0.3746, *P* = 0.5425), extinction (Day 1: F (1, 84) = 8.873, *P* = 0.0038; Day 2: F (1, 84) = 4.522, *P* = 0.0364) and extinction retrieval (F (1, 84) = 10.88, *P* = 0.0014), two-way repeated measures ANOVA. (**F**) Freezing responses during fear renewal, t (14) = 0.2451, *P* = 0.8099, unpaired *t* test. Data are presented as means ± SEM, **P* < 0.05, ***P* < 0.01.


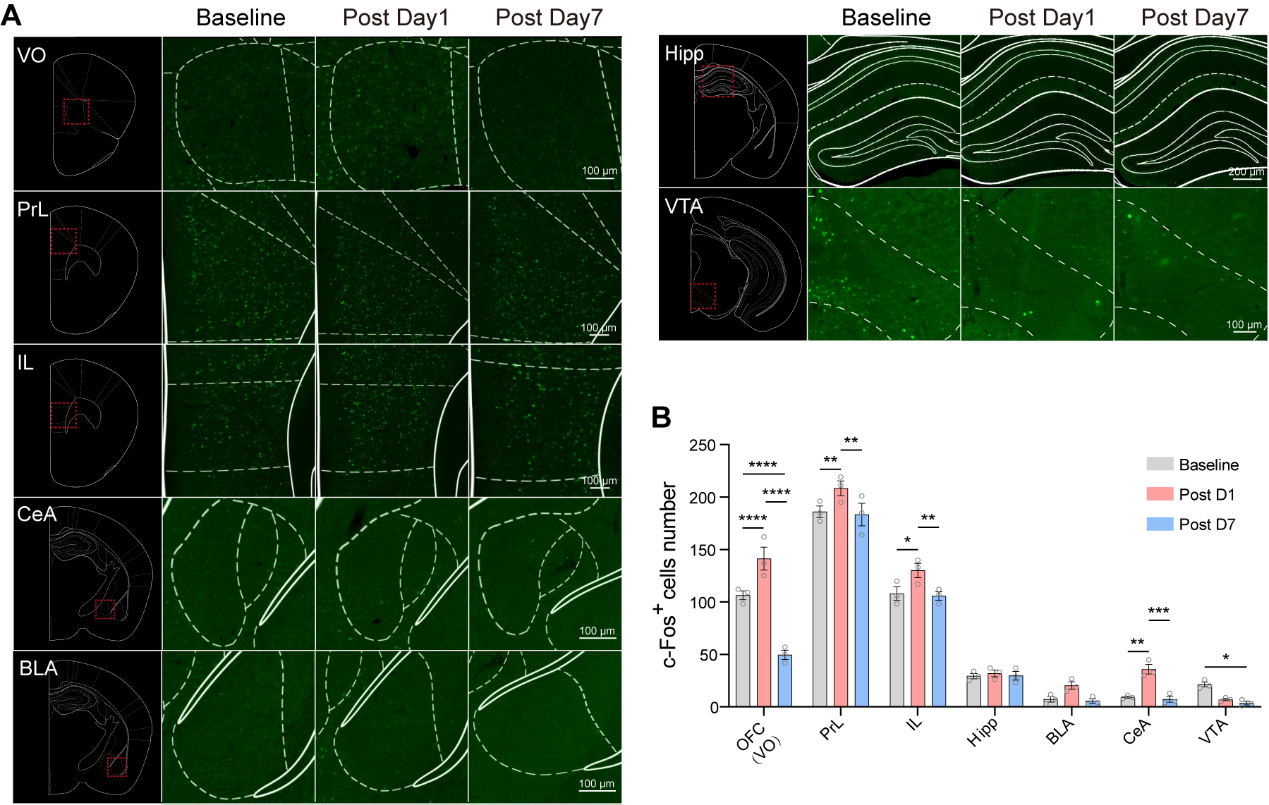


**Fig.S5. Temporal changes of c-Fos expression in PTSD-associated brain regions at 1 day and 7 days after SPS&S modeling.** (**A**) Representative coronal immunofluorescence images of c-Fos expression across brain regions: ventral orbital cortex (VO), prelimbic cortex (PrL), infralimbic cortex (IL), central amygdaloid nucleus (CeA), basolateral amygdala (BLA), hippocampus (HPC), and ventral tegmental area (VTA). (**B**) Quantification of c-Fos-immunoreactive (c-Fos+) cells. F (2, 42) = 49.14, *P* < 0.0001, two-way repeated measures ANOVA. *n* = 3, Data represent mean ± SEM, * *P* < 0.05, ** *P* < 0.01, *** *P* < 0.001, **** *P* < 0.0001.


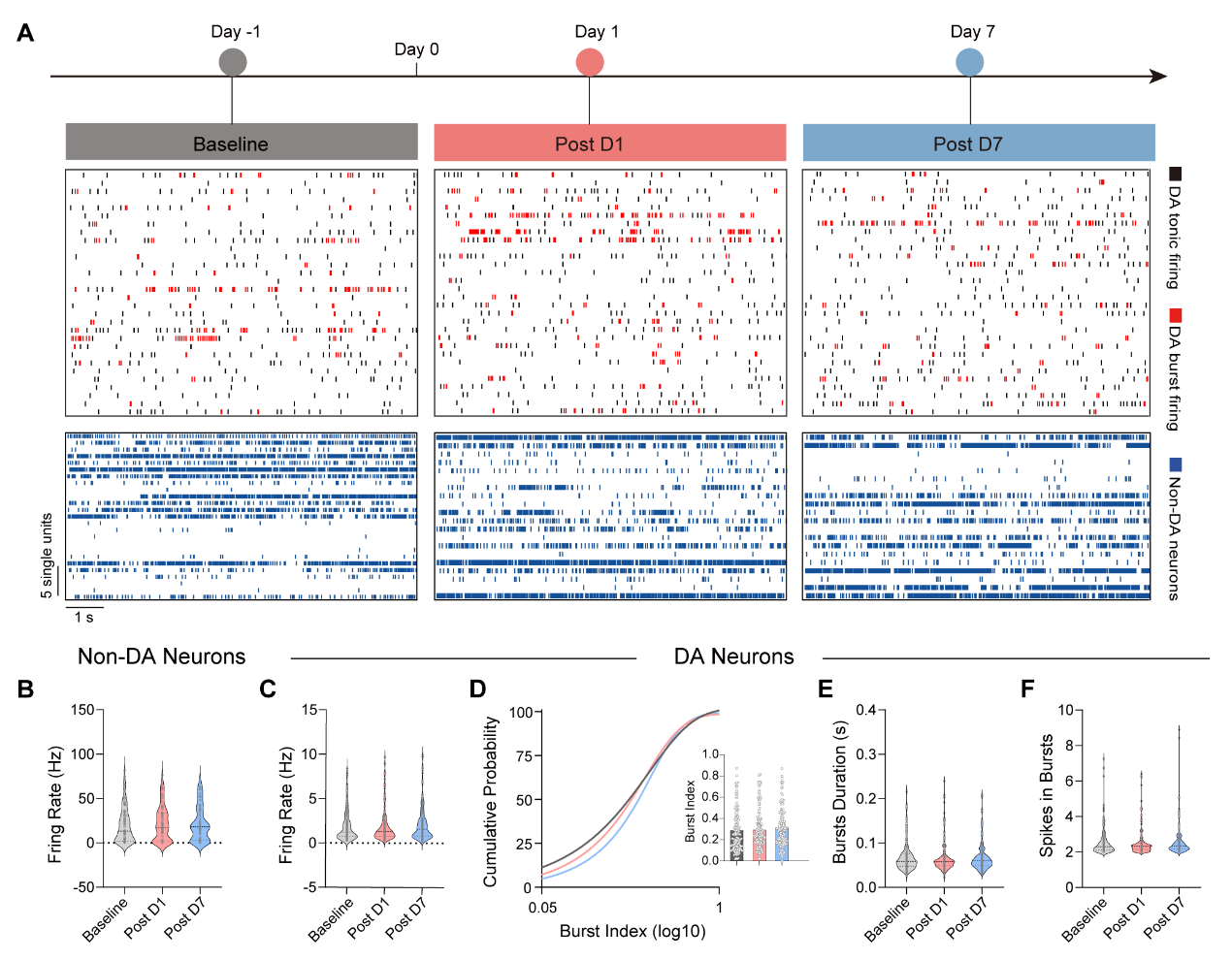


**Fig. S6. Unchanged firing properties of VTA neurons over time following chronic electrode implantation.** (**A**) Schematic of in vivo electrophysiology recording (top) and representative raster plot of DA and non-DA neurons. (**B**) Firing rate of non-DA neurons, *P* = 0.7994, Kruskal-Wallis test, *n* = 79 units in Baseline, *n* = 67 units in Post D1, *n* = 66 units in Post D7. (**C**) Firing rate of DA neurons (*P* = 0.3014). (**D**) Cumulative distribution and comparison of the burst index (the ratio of burst events to the total number of spikes) of DA neurons; *P* = 0.1697. (**E-F**) Bursts duration (*P* = 0.7040) and spikes in bursts (*P* = 0.4846) of DA neurons, Kruskal-Wallis test, DA neurons *n* = 140 units in Baseline, *n* = 165 units in Post D1, *n* = 147 units in Post D7. Data are presented as means ± SEM.


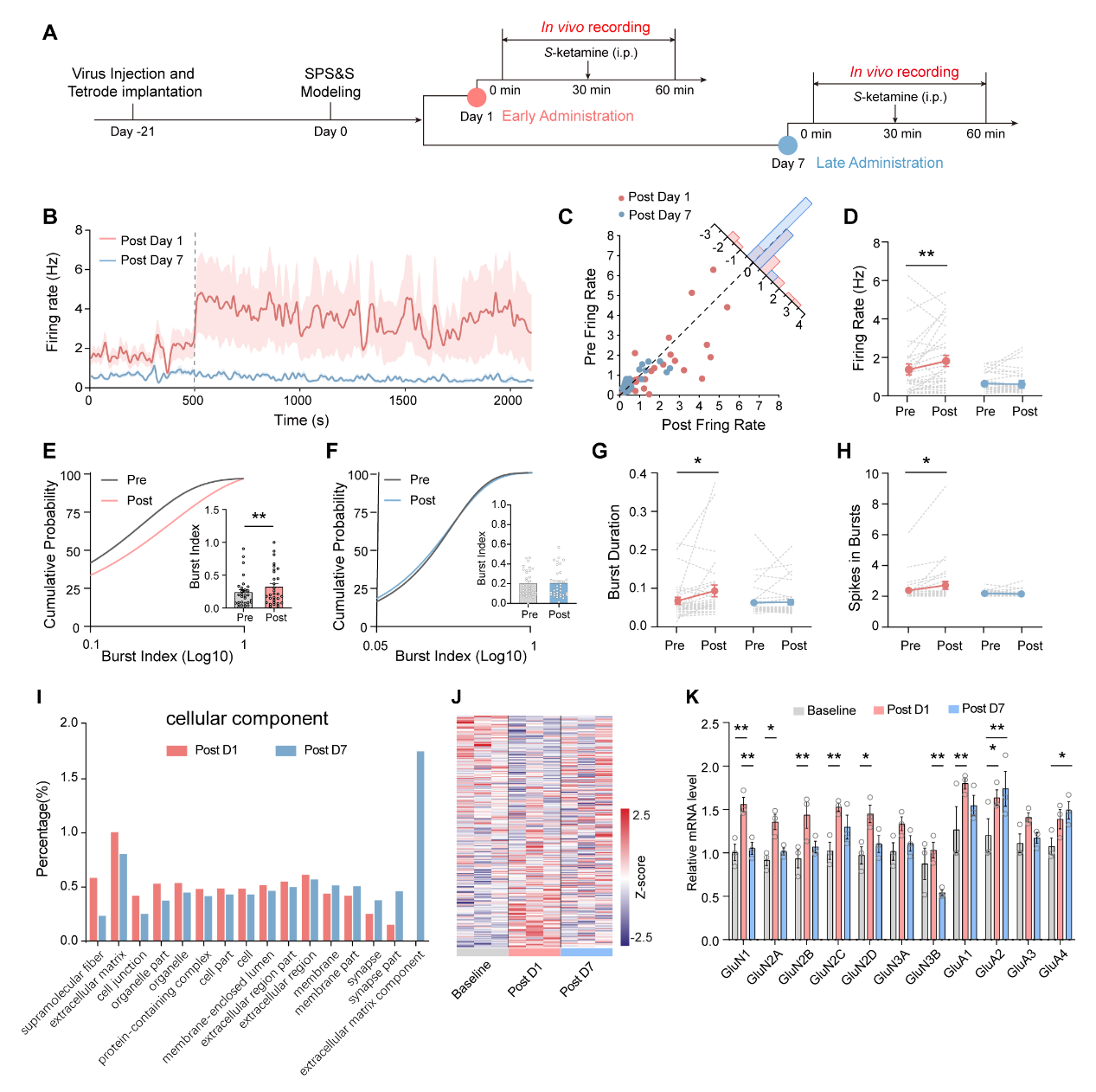


**Fig.S7. Early rather late administration of *S*-ketamine increases VTA^DA^ neurons excitability promptly and VTA synaptic plasticity.** (**A**) Schematic of in vivo electrophysiology recording. (**B-D**) Firing rate of DA neurons in VTA before and after intraperitoneal injection of *S*-ketamine (10 mg/kg), Post Day 1 (*P* = 0.0087, W = 251, *n* = 30), Post Day 7 (*P* = 0.1028, W = -200, *n* = 35), *Wilcoxon* test. (**E**) Cumulative distribution and comparison of the burst index (the ratio of burst events to the total number of spikes) of Post Day 1, t (29) = 2.809, *P* = 0.0088, *n* = 30, Paired *t* test. (**F**) Cumulative distribution and comparison of the burst index (the ratio of burst events to the total number of spikes) of Post Day 7, W = -150, *P* = 2254, *n* = 35, *Wilcoxon* test. (**G**) Bursts duration (Post Day 1: W = 211, *P* = 0.0293, *n* = 30; Post Day 7: W = 266, *P* = 0.2162, *n* = 35), *Wilcoxon* test. (**H**) Spikes in bursts (Post Day 1: W = 203, *P* = 0.0194, *n* = 30; Post Day 7: W = -346, *P* = 0.0004, *n* = 35), *Wilcoxon* test. (**I**) Enrichment results of two groups of 232 DEGs in cellular components. (**J**) The heat map showing the gene. (**K**) Relative mRNA levels of subunit of NMDA and AMPA receptors in VTA brain regions of three groups, *n* = 3, F (2,66) = 37.19, *P* < 0.0001. two-way ANOVA. Data are presented as means ± SEM, **P* < 0.05, ***P* < 0.01.


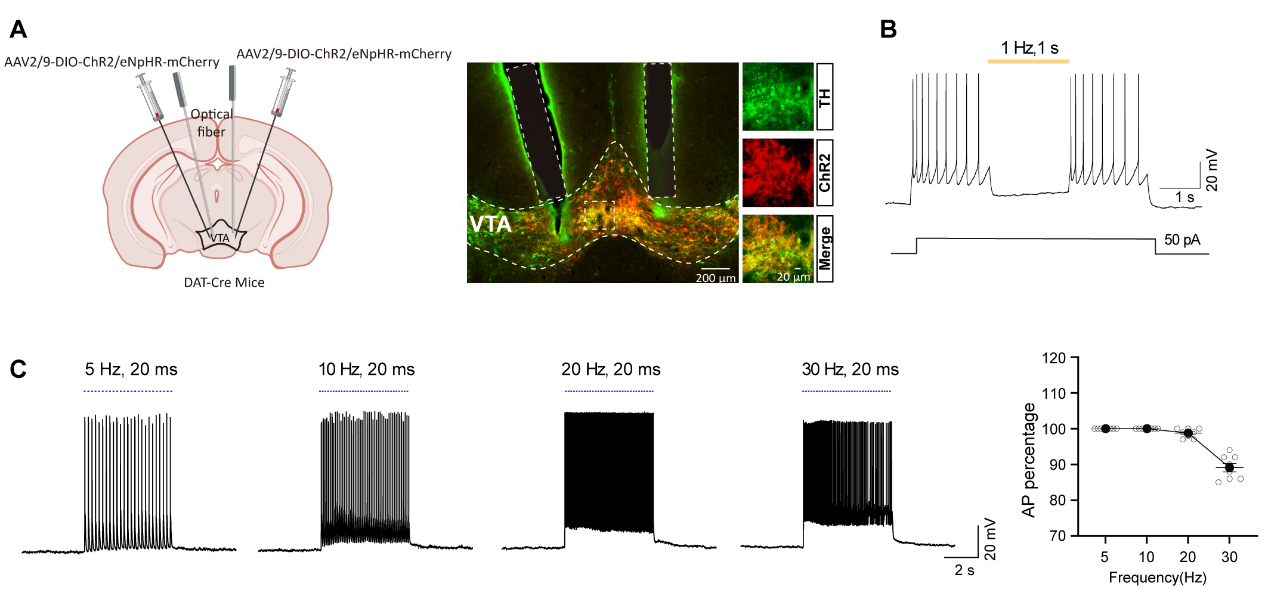


**Fig. S8. Validation of optogenetic manipulation in VTA^DA^ neurons.** (**A**) Schematic of optical fiber implantation, viral injection (left) and representative image of viral expression and localization of fiber in the VTA (right). (**B**) Raw traces showing action potentials of VTA^DA^ neurons inhibited by 1 Hz 594 nm laser stimulation. (**C**) Raw traces showing action potentials of VTA^DA^ neurons evoked by 5, 10, 20, 30 Hz 473 nm laser stimulation and quantification of the percentage of laser-evoked action potentials. *n* = 8 neurons from 4 mice.


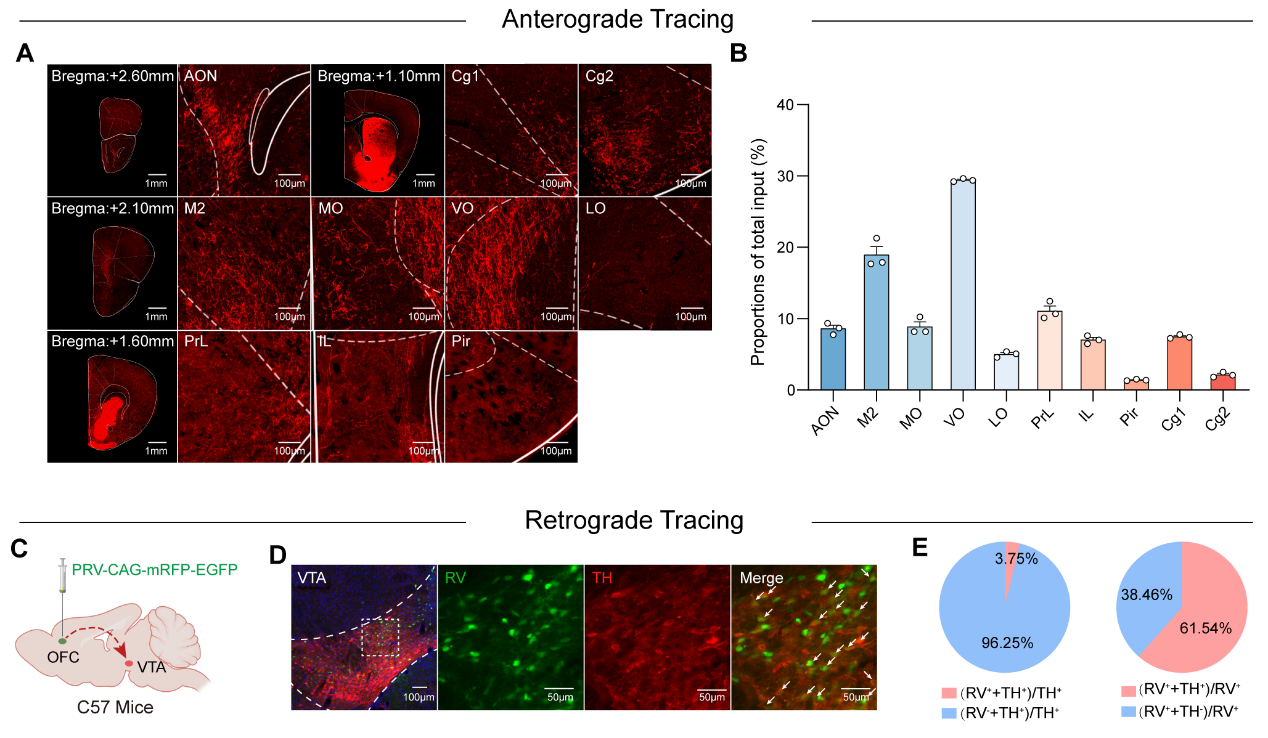


**Fig.S9. Comprehensive projection mapping and quantitative analysis of VTA^DA^ neuronal connectivity with the frontal cortex.** (**A**) Representative coronal sections of the frontal cortex showing axonal projections from VTA^DA^ neurons. Abbreviations: AON, anterior olfactory nucleus; M2, secondary motor cortex; MO, medial orbital cortex; VO, ventral orbital cortex; LO, lateral orbital cortex; PrL, prelimbic cortex; IL, infralimbic cortex; Pir, piriform cortex; Cg1, cingulate cortex area 1; Cg2, cingulate cortex area 2. (**B**) Proportion of total axonal arborization from VTA^DA^ neuron projections in the frontal cortex. (**C**) Schematic of virus injection to retrograde tracing. (**D**) Representative OFC retrograde tracing in VTA. (**E**) Quantification of retrograde tracing results from the OFC to the VTA. *n* = 3.


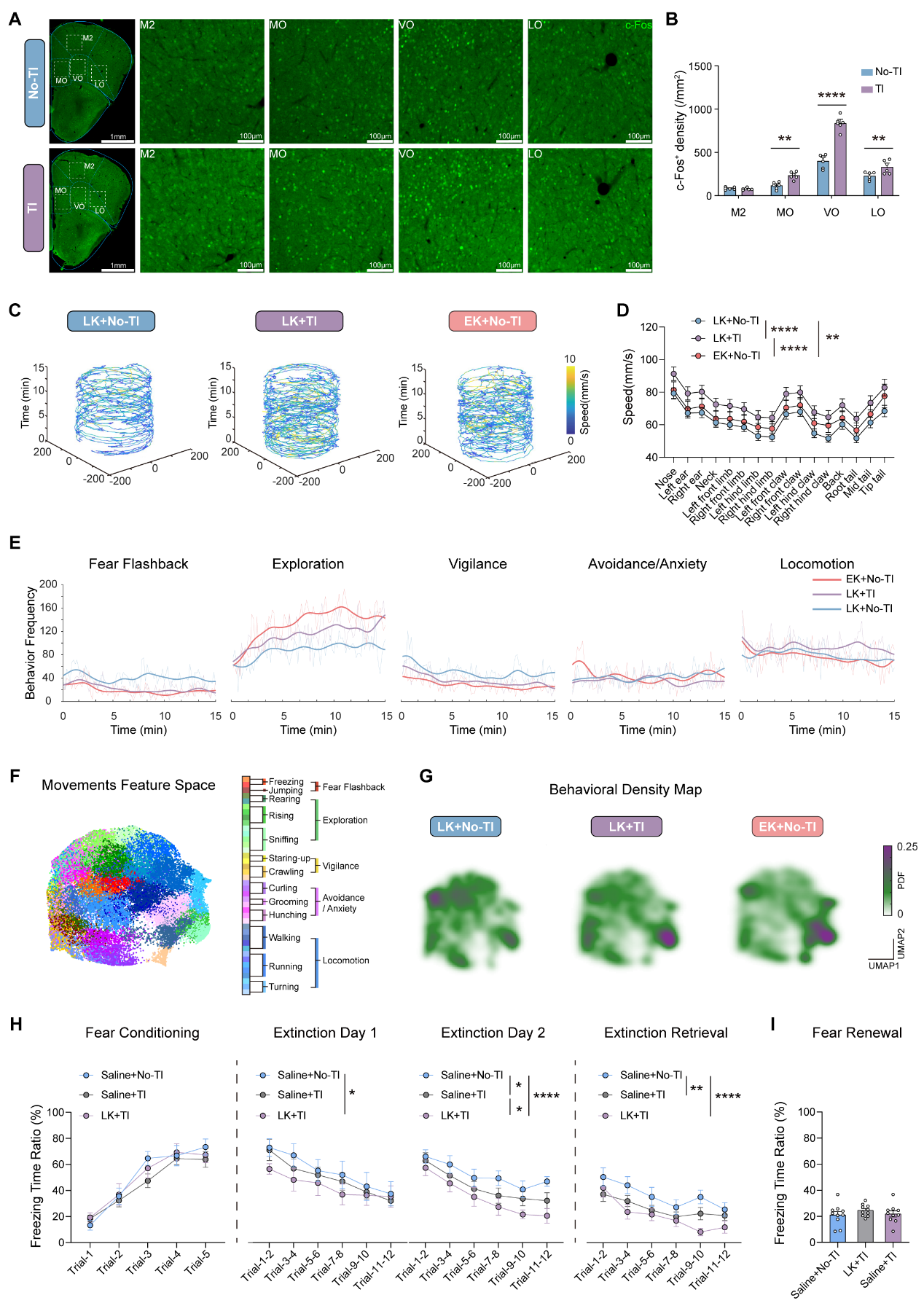


**Fig.S10. Spontaneous behavioral variation after *S*-ketamine intervention combined with TI-NIBS sensitizing.** (**A**) Representative images showing c-Fos expression in No-TI and TI groups. (**B**) c-Fos^+^ density of secondary motor cortex (M2), medial orbital cortex (MO), ventral orbital cortex (VO), lateral orbital cortex (LO), F (2, 66) = 37.19, *P* < 0.0001, *n* = 3 mice, two-way ANOVA. (**C**) Representative of speed dynamics. (**D**) The speed of 16 body points across three groups of mice. F (2, 448) = 38.32, *P* < 0.0001, two-way ANOVA. (**E**) Frequency of five behavioral categories among four groups. (**F**)Low-dimensional visualization of 40 movements generated through UMAP dimensionality reduction. (**G**) Heatmap of the feature space probability density of each group of behavior segments. Areas with higher probability density (purple) indicate denser behavioral segments in that region, while areas with lower probability density (white) indicate sparser behavioral segments. (H) Freezing responses to the CS during fear conditioning (F (2, 135) = 1.262, *P* = 0.2863), extinction (Day 1: F (2, 162) = 3.157, *P* = 00452; Day 2: F (2, 162) = 12.88, *P* < 0.0001) and extinction retrieval (F (2, 162) = 13.54, *P* < 0.0001), two-way repeated measures ANOVA. (I) Freezing responses during fear renewal, F (2, 27) = 0.4764, *P* = 0.4847, one-way ANOVA with Tukey test. Saline+No-TI, *n* = 10 mice; Saline+TI, *n* = 10 mice; LK+TI, n = 10 mice. Data are presented as means ± SEM, **P* < 0.05, ***P* < 0.01, *****P* < 0.0001. LK+No-TI, *n* = 11 mice, LK+ TI, *n* = 10 mice, EK+No-TI, *n* = 10 mice, Saline+No-TI, *n* = 10 mice, Saline+TI, *n* = 10 mice. Data are presented as means ± SEM, **P* < 0.05, ***P* < 0.01, ****P* < 0.001, *****P* < 0.001. PDF, probability density function.


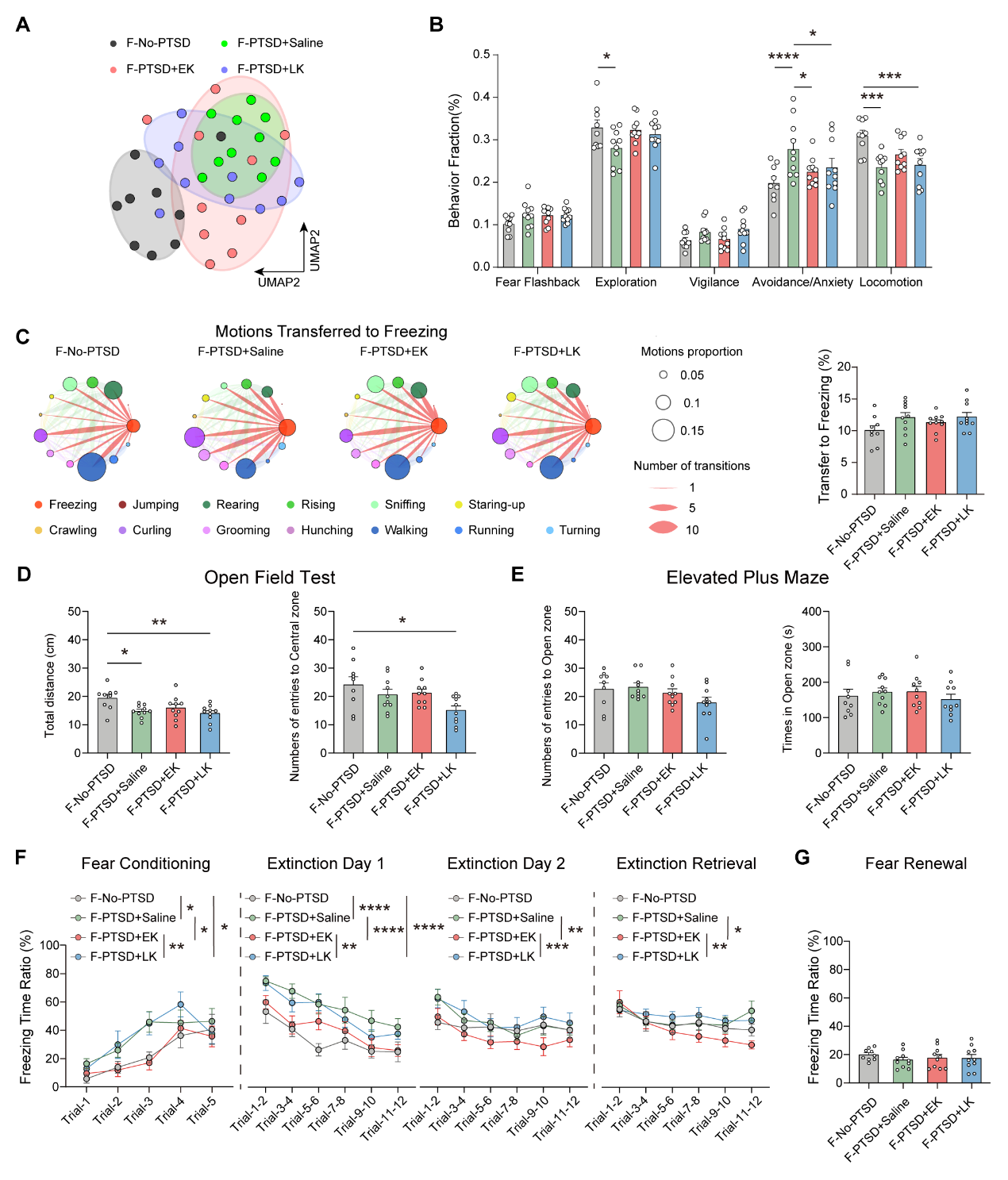


**Fig.S11. Time-dependent therapeutic effects of *S*-ketamine in female mice.** (**A**) Low-dimensional UMAP representation of the female mice among different groups based on spontaneous behaviors. (**B**) The fraction of five behavioral categories, one-way ANOVA. (**C**) Movements transferred to freezing. The size of the colored circle represents the proportion of the corresponding behavior, and the diameter of the line segment represents the number of transformations. F (3, 35) = 0.8469, *P* = 0.13, one-way ANOVA with *Tukey* test. (**D**) Behavioral performance in open field test. Total distance traveled (left, F (3, 35) = 0.8236, *P* = 0.0084), numbers of entries to center zone (right, F (3, 35) = 1.339, *P* = 0.0205), one-way ANOVA with *Tukey* test. (**E**) Behavioral performance in elevated plus maze test. Numbers of entries to open zone (left, *Kruskal-Wallis* statistic = 5.120, *P* = 0.1632, *Kruskal-Wallis* test) and times in open zone (right, F (3, 35) = 0.297, *P* = 0.4728, one-way ANOVA with *Tukey* test). (**F**) Freezing responses to the CS during fear conditioning (F (3, 175) = 6.117, *P* = 0.0006), extinction (Day 1: F (3, 204) = 19.12, *P* < 0.0001; Day 2: F (3, 204) = 6.058, *P* = 0.0006), and extinction retrieval (F (3, 204) = 4.514, *P* = 0.0043), two-way repeated measures ANOVA. (**G**) Freezing responses during fear renewal, F (3, 35) = 1.321, *P* = 0.5030, one-way ANOVA with *Tukey* test. F-NO-PTSD, *n* = 9 mice, F-PTSD+Saline, F-PTSD+EK, F-PTSD+LK, *n* = 10 mice. Data are presented as means ± SEM, **P* < 0.05, ***P* < 0.01, ****P* < 0.001, *****P* < 0.0001. PDF, probability density function; UMAP, uniform manifold approximation and projection.


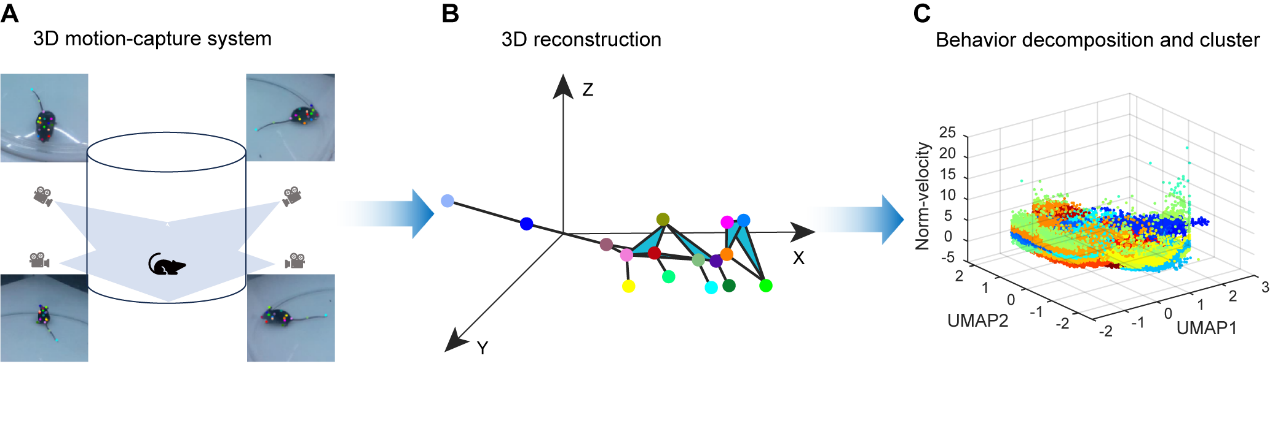


**Fig. S12. The specific process of 3D-AI behavior analysis.** (**A**) Synchronous collection of multi-angle experimental animal videos. (**B**) Reconstruction of the three-dimensional skeleton of experimental animals according to body point tracking data. (**C**) Segmentation of mouse behavior segments based on the 3D skeleton and clustering of behavioral segments.

**Supplementary Table S1 Definition of the behaviors (ethogram) for manual labeling**

| **Category Behavior** | **Definition** |
| --- | --- |
| Fear Flashback  Freezing | The mouse complete immobility and stillness. |
| Jumping | The mouse leaps into the air using the rapid contraction of its hind legs and its body vertical or nearly vertical. |
| Exploration  Rearing | The mouse stands on its hind legs, and the back is straight. |
| Rising | The mouse rises from four legs on the ground to steadily stand on its hind legs. |
| Sniffing | The mouse investigates environment with the nose held in the air or contacts the environment with nose closely. |
| Vigilance  Staring-up | The mouse remains stationary with head-raising and staring. |
| Crawling | The mouse remains stationary with its limbs extended outward and its head raised to observe its surroundings |
| Avoidance/Anxiety  Curling | The mouse curls up its body. |
| Grooming | The mouse clicks its fur, grooms with the forepaws, or scratches with any limb. |
| Hunching | The mouse stands on its hind legs while the back is bent. |
| Locomotion  Walking | The mouse locomotes with relatively low speed. |
| Running | The mouse locomotes with relatively high speed. |
| Turning | The mouse bends its body to right/left or turns body to right/left while walking. |
